# Investigating Learning, Decision-Making, and Mental Health in Pregnancy: Insights From a UK Cohort Study

**DOI:** 10.5334/cpsy.134

**Published:** 2025-09-03

**Authors:** Ilaria Costantini, Axel Montout, Paul Moran, Daphne Kounali, Rebecca M. Pearson, Casimir J. H. Ludwig

**Affiliations:** 1 Centre for Academic Mental Health, University of Bristol, Oakfield House, Bristol, United Kingdom; 2 School of Psychological Science, University of Bristol, Bristol, United Kingdom; 3 Division of Psychiatry, University College London, London, United Kingdom; 4 Bristol Veterinary School, University of Bristol, Bristol, United Kingdom; 5 NIHR Biomedical Research Centre at the University Hospitals Bristol NHS Foundation Trust, Bristol, United Kingdom; 6 Population Health Sciences, Bristol Medical School, University of Bristol, Bristol, United Kingdom; 7 Bristol NIHR Biomedical Research Centre, Bristol, United Kingdom; 8 Oxford Clinical Trials Research Unit, Centre for Statistics in Medicine, Nuffield Department of Orthopaedics, Rheumatology and Musculoskeletal Sciences, University of Oxford, Oxford, United Kingdom; 9 Faculty of Health and Education, Manchester Metropolitan University, Manchester, United Kingdom

**Keywords:** ALSPAC, bandit-task, antenatal depression and personality difficulties, reference point, learning and decision-making processes

## Abstract

**Background:**

Parental capacity to learn from infant responses is a fundamental component of early dyadic interactions. However, the precise cognitive processes involved in these interactions and how these processes are influenced by mental health difficulties remain unclear.

**Methods:**

We investigated the computational basis of learning and decision-making in males and nulliparous females (Study 1) and pregnant participants enrolled in a cohort study (Study 2), using a two-armed bandit task adapted to simulate playful interactions with an infant. Participants chose between two competing bandits (i.e., two toys) with different underlying nominal probabilities for three outcomes (i.e., infant sad, neutral, and happy facial expressions). In Study 1, we manipulated the baseline emotional context of the task (i.e., the infant started either happy or sad) to investigate its effect on the processing of emotional feedback and decision-making. In both studies, we explored whether individual differences in mental health and personalities difficulties associated with variation in parameters.

**Results:**

In Study 1, the emotional context manipulation influenced both learning rates and how neutral outcomes were evaluated. Participants starting with a happy infant exhibited faster learning and a more negative evaluation of neutral outcomes compared to those starting with a sad infant. In Study 2, participants reporting higher levels of personality difficulties and antenatal depressive symptoms showed reduced learning rates. These associations were weaker in Study 1.

**Conclusions:**

Our findings provide novel evidence regarding the role of the emotional context in learning and decision-making processes. For parents with depressive symptoms and personality difficulties, dampened responsivity to emotional feedback and inflexibility in updating beliefs about the values of actions may underlie fewer sensitive behaviours when interacting with their infants.

## Introduction

The quality of parent-infant interactions is a putative risk factor for child emotional problems (Cooke et al., 2022; Dagan et al., 2021). Poorer maternal mental health, personality difficulties, and more difficult child temperament have been linked to increased difficulties in such interactions as well as less optimal emotional outcomes in children (Bornstein et al., 2021; Eyden et al., 2016; Goodman et al., 2020; Høivik et al., 2018; Ivanova et al., 2022; Nath et al., 2020; Pearson et al., 2018). However, the cognitive processes underpinning early mother–infant interactions – which could offer mechanistic insights and novel targets to enhance interventions – and how these processes vary in relation to mental health symptoms, remain poorly understood.

Understanding how individuals learn from infant-related feedback represents an important area for investigation given the relevance of social cognition in parenthood (Hoekzema et al., 2017; King et al., 2021; Parsons et al., 2017) and how it can be affected by mental health difficulties (Barba-Müller et al., 2019; De Carli et al., 2019). Starting in pregnancy, women undergo profound neurobiological changes, including reorganisation of brain regions supporting social cognition and emotional processing (Cárdenas et al., 2020; Hoekzema et al., 2017). These adaptations have been proposed to support caregiving behaviour (Anderson & Rutherford, 2012), although evidence is mixed and often strong causal claims are hindered by limitations in study designs (Humphreys & Kujawa, 2024).

From a cognitive perspective, caregiving can be conceptualised as a learning process. For instance, during a playful interaction, a caregiver may learn through trial and error which actions (e.g., toy selection) elicit positive infant responses (e.g., smiling). However, as infant preferences are dynamic, successful caregiving requires not only exploiting known rewarding behaviours but also exploring alternative strategies. This trade-off between exploiting known rewards and exploring new options is classically formalised as the “exploration–exploitation dilemma” in reinforcement learning (RL).

Multi-armed bandit tasks provide a well-established framework to study such learning processes (Sutton & Barto, 2018). In these tasks, an agent selects among different options (bandits) to maximise rewards over time. Computational models fitted to choice data typically estimate a learning rate (α), which reflects how rapidly value estimates are updated following feedback, and an inverse temperature (τ), which captures the stochasticity of decision-making, or the balance between exploration and exploitation.

However, social context may modulate the evaluation of outcomes and thus influencing both learning and decision-making processes (Bavard et al., 2018; Vandendriessche et al., 2021). According to Prospect Theory (Tversky & Kahneman, 1979), outcomes are judged relative to a reference point, and this reference point is context-dependent (Palminteri et al., 2015; Palminteri & Lebreton, 2021). Applied to our caregiving scenario, an infant’s baseline emotional state (e.g., crying or smiling) may serve as a contextual reference, influencing how neutral responses are experienced and valued and what type of decisions (e.g., explorative or exploitative) are favoured.

Difficulties in learning and decision-making processes under uncertainty are common in various mental health conditions. Individuals with anxiety symptoms often show an over-sensitivity to ambiguous stimuli and uncertain situations (Grupe & Nitschke, 2013), which may lead to subsequent behaviours aimed at avoiding or reducing uncertainty (Huang et al., 2017; Lamba et al., 2020). Conversely, high levels of impulsivity have been linked to greater risk-taking and tolerance of uncertainty (Krmpotich et al., 2015). Altered learning from feedback, particularly diminished use of external information to update knowledge, has been observed in individuals suffering from anxiety and mood disorders (Aylward et al., 2019; Eshel & Roiser, 2010; Halahakoon et al., 2020). Similarly, individuals with personality difficulties, including borderline personality disorder, often show rigid learning patterns and impaired adaptation to social feedback (Henco et al., 2020; Paret et al., 2017; Unoka & Richman, 2016).

In the present study, we developed a novel two-armed bandit task specifically designed to simulate a playful caregiving interaction with an infant. Unlike conventional reinforcement learning paradigms, our task incorporated emotionally salient stimuli (images of crying, neutral, or happy infants) and a reward structure that mimicked caregiving goals (soothing or maintaining a baby’s positive emotional state). A model-based analysis was then used to characterise participants’ learning and decision-making processes.

Since pregnancy and parenting may influence these processes, we first assessed the influence of baseline emotional context in a nulliparous sample (Study 1), where the salience of infant stimuli would be less variable due to the absence of maternal experience. However, we acknowledge that the extent to which pregnancy-related changes facilitate caregiving behaviour remains debated (Cárdenas et al., 2020; Humphreys & Kujawa, 2024).

We formulated two sets of hypotheses addressing distinct levels of analysis: task-related effects and individual differences.

First, we hypothesised a task-related effect. Specifically, we expected that the utility of the neutral outcome (i.e., how participants interpret and use the neutral feedback to guide their next choice) would depend on the baseline emotional context. We hypothesised that participants starting with a crying baby would experience the neutral outcome more positively compared to participants starting with a happy baby. In addition, we hypothesised that the baseline emotional context would influence decision-making strategies, with participants starting with a crying baby showing more exploratory behaviour compared to those starting with a happy baby.

Second, we hypothesised that participants with higher scores on anxiety measures would be faster in learning from feedback – e.g., reflecting heightened sensitivity to (negative) feedback (Aylward et al., 2019) and that participants with higher levels of depressive symptoms or personality difficulties would exhibit lower learning rate, driven by lower sensitivity to rewarding stimuli and greater inflexibility in updating their strategies, respectively (Mukherjee et al., 2020).

## Methods

### Participants Study 1

203 participants (85.4% self-identified women, mean [SD] age in years = 19.82 (2.73)) were recruited using the University of Bristol (UoB) psychology undergraduate participant pool, student and staff email lists, and word-of-mouth. Psychology undergraduate students were given course credits for taking part in the study. Inclusion criteria required that participants were 18–50 years of age, fluent in English, and either nulliparous (i.e., females who had never experienced a pregnancy) or, for males, never to have experienced parenthood. We did not exclude participants based on identified gender. Participants were given an information sheet and task instructions before completing the task. The School of Psychological Science Research Ethics Committee approved the study (Ethical approval code: 28031983924). All participants provided written informed consent at the start (i.e., to take part in the study) and at the end (i.e., for data usage) of the experiment. Participants were excluded from the analyses if they chose the same bandit for more than 75% of the trials, indicating poorer learning than chance or disengagement in the task by clicking through the entire experiment on the same response (*n* excluded = 12 (6% attrition); *n* of included students in the analyses = 191). Participants were randomly assigned to one of the two emotional context conditions (“Soothe the baby” – on each trial participants were shown a crying baby face – or “Keep the baby happy” – on each trial participants were shown a happy baby face). Descriptive statistics by randomised group are presented in Table 1. In accordance with CONSORT guidelines, no formal statistical comparisons of baseline demographic or psychological characteristics were conducted between groups.

**Table 1:** Basic demographic characteristics of participants in Study 1, by randomised condition. Participants were randomly assigned to one of the two emotional context conditions (“Soothe the baby” – on each trial participants were shown a crying baby face – or “Keep the baby happy” – on each trial participants were shown a happy baby face).


CHARACTERISTIC	‘SOOTHE THE BABY’ N = 103	‘KEEP THE BABY HAPPY’ N = 100

Age	19.70 (2.43)	19.88 (2.89)

**Ethnicity**		

White or Caucasian	83 (81%)	82 (83%)

Asian or Asian American	14 (14%)	9 (9.1%)

Other ethnicity	6 (5.8%)	8 (8.1%)

**Gender**		

Girl	90 (87%)	82 (83%)

Boy	12 (12%)	17 (17%)

Prefer not to say	1 (1.0%)	0 (0%)

**Highest Education**		

Below undergraduate	8 (7.8%)	3 (3.0%)

Undergraduate or higher	95 (92%)	97 (97%)

**Income**		

Under £15,000	9 (11%)	11 (13%)

£15,000–£29,999	19 (22%)	17 (21%)

£30,000–£49,999	17 (20%)	14 (17%)

£50,000–£74,999	14 (16%)	15 (18%)

£75,000–£99,999	8 (9.4%)	11 (13%)

Over £100,000	18 (22%)	14 (17%)

**Age of Menarche**		

9–10 years	9 (10%)	6 (7.3%)

11–12 years	40 (44%)	33 (40%)

13–14 years	34 (38%)	35 (43%)

15+ years	7 (7.8%)	8 (9.8%)

**Hormonal Contraceptive**		

Yes	49 (54%)	44 (54%)

No	41 (46%)	38 (46%)


*Note*. Other ethnicity includes Black, Hispanic, and other groups. Education categories were combined into ‘Below undergraduate’ and ‘Undergraduate or higher’ due to small sample sizes. Income categories over £100,000 were merged into ‘Over £100,000’.

### Participants Study 2

Data were obtained from participants of the Avon Longitudinal Study of Parents and Children (ALSPAC), also known as Children of the 90s, which is an ongoing prospective population-based birth cohort study. Between 1990 and 1992, all pregnant women residing in Bristol and the surrounding area were invited to take part in ALSPAC. 14,541 pregnant women were recruited. The original mothers and partners [Generation 0: ALSPAC-G0 (Fraser et al., 2013)], and their living children [Generation 1: ALSPAC-G1 (Boyd et al., 2013; Northstone et al., 2019)], have been followed-up regularly since recruitment through questionnaires and clinic assessments. Ethical approval for the study was obtained from the ALSPAC Law and Ethics Committee and Southwest National Health Service (NHS) Research Ethics Committee; participants gave written informed data consent. In 2012, the recruitment of the second generation of ALSPAC (ALSPAC-G2) (Lawlor et al., 2019) started: the aim was to recruit all the children of ALSPAC-G1 as well as to recruit all the (non-ALSPAC) partners of the ASLPAC-G1 parents. Data were collected from both parents (at least one of whom is a G1 participant) and their children. Study data were collected and managed using REDCap electronic data capture tools hosted at the University of Bristol (Harris et al., 2009). REDCap (Research Electronic Data Capture) is a secure, web-based software platform designed to support data capture for research studies. The study website contains details of all data available through a fully searchable data dictionary (http://www.bristol.ac.uk/alspac/researchers/our-data/).

**Figure 1:**
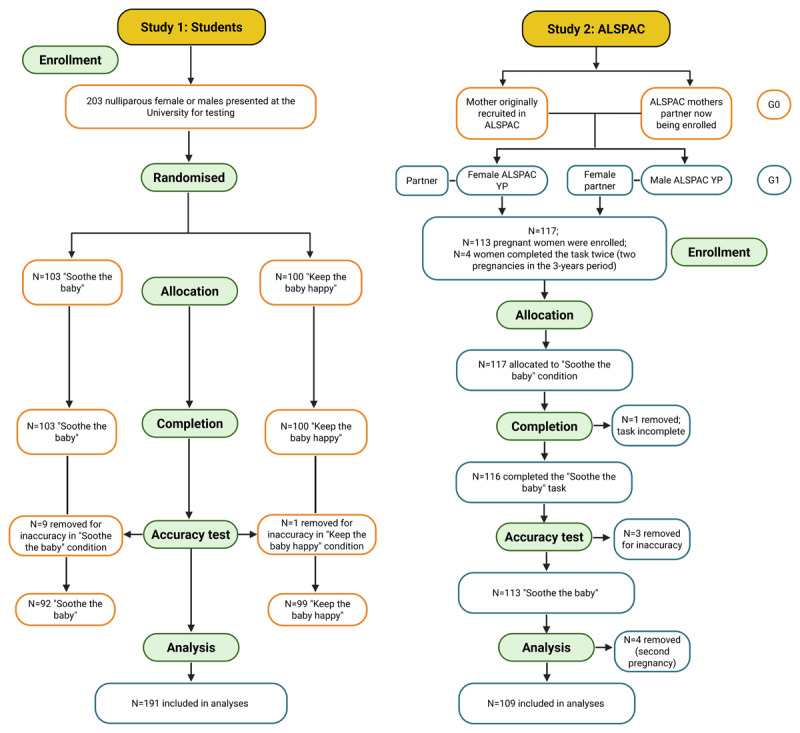
Participant recruitment, allocation and attrition/removal in Study 1 and Study 2. *Note*. This figure was created in BioRender. Costantini, I. (2025) https://BioRender.com/7ppxkl9.

The sample included both women pregnant with their first child (primiparous) and those who were already parents (multiparous). 150 participants were invited to complete the task during the clinic visit at 18 weeks of gestation (even though participants could have attended later) (Lawlor et al., 2019). 129 (86%) consented to take part in the data collection, of these 117 (91%) completed the task, with 4 participants completing the task twice (one per pregnancy) (*n* unique = 112). 28 (24%) tasks were completed during the in-person clinic visit using Psychopy 3.0 coder (Peirce et al., 2019) on a computer desktop (dates: October 2019 – March 2020; with *n* = 3 in October 2021), whereas the remaining were completed during Covid lockdowns and were collected online using Gorilla Experiment Builder (Anwyl-Irvine et al., 2020) (www.gorilla.sc). A separate Ethics application and risk assessment was conducted before collecting online data (dates of collection: May 2021 – September 2022). One participant was removed because they did not complete the task (trials completed = 62 out of 200). Participants were excluded from the analyses according to our accuracy criterion (*n* = 3) and when the task was completed a second time (*n* = 4) (N of included ALSPAC participants in the analyses = 109). 91 (83%) out of 109 participants included in the analyses were ALSPAC G1 YP (i.e., Young Person), whilst 18 were partners of an ALSPAC-G1 YP. A flow chart describes ALSPAC-G1 and non-ALSPAC participants included in these analyses (Figure 1).

It is important to note that in study 2, ‘pregnant women’ refers to participants who were pregnant at the time of testing and assigned female at birth; however, data on gender identity were not collected, so this term may not reflect participants’ self-identified gender.

### Materials

#### Questionnaires

In Study 1, participants completed questionnaires on a computer using Qualtrics (Qualtrics, 2005). We collected basic demographic information including gender, age, socio-economic status, education, and ethnicity. In addition, participants completed the following standardised mental health measures:

- **State-Trait Anxiety Inventory (STAI-Form Y1 and Y2)** (Spielberger, 1983): a 40-item self-report instrument used to assess state and trait anxiety levels. Scores range from 20 to 80 for each subscale, with higher scores indicating greater anxiety. Internal consistency in the present sample was high (Cronbach’s α = 0.93 for both subscales).
- **Short-Mood and Feeling Questionnaire (SMFQ)** (Messer et al., 1995): a 13-item measure assessing depressive symptoms in young adults. Each item is scored from 0 (“not true”) to 2 (“true”), with total scores ranging from 0 to 26. Higher scores indicate greater depressive symptom severity. Internal consistency in the present sample was high (Cronbach’s α = 0.86).
- **Standardised Assessment of Personality–Abbreviated Scale (SAPAS)** (Moran et al., 2003): an 8-item screening tool designed to assess core features of personality disorder, such as impulsivity, mistrust, and emotional instability. Responses are binary (yes/no), with higher scores indicating greater personality difficulties. In the present study, we use the term ‘personality difficulties’ to refer to enduring patterns of cognition, affect, and behaviour. Internal consistency in Study 1 was modest (Cronbach’s α = 0.47) but consistent with previous studies (Messer et al., 1995).
- **Barratt Impulsiveness Scale 11 (BIS-11)** (Barratt, 2007): a 30-item self-report questionnaire evaluating attentional, motor, and non-planning impulsivity. Items are rated on a 4-point scale (1 = rarely/never to 4 = almost always/always). Higher scores indicate higher impulsivity. Internal consistency in the current sample was good (Cronbach’s α = 0.80).

In Study 2 (pregnant participants from the ALSPAC-G1 cohort and their partners), the following measures were completed:

- **Edinburgh Postnatal Depression Scale (EPDS)** (Murray & Cox, 1990) (*n* = 80, 73% of total sample). The EPDS is a validated 10-item self-report measure designed to screen women for depression both during and after pregnancy. Scores range from 0 to 30, with higher scores indicating more severe symptoms. Cronbach’s alpha in the present sample was 0.72.
- **Standardised Assessment of Personality – Abbreviated Scale (SAPAS)** (Moran et al., 2003) (*n* = 91, 83% of the total sample): used again to measure personality difficulties (Cronbach’s α = 0.58)

Although BIS-11 data were collected in Study 2, these were not analysed due to over 70% missingness, and the absence of suitable auxiliary variables for imputation.

#### Bandit task

**Figure 2:**
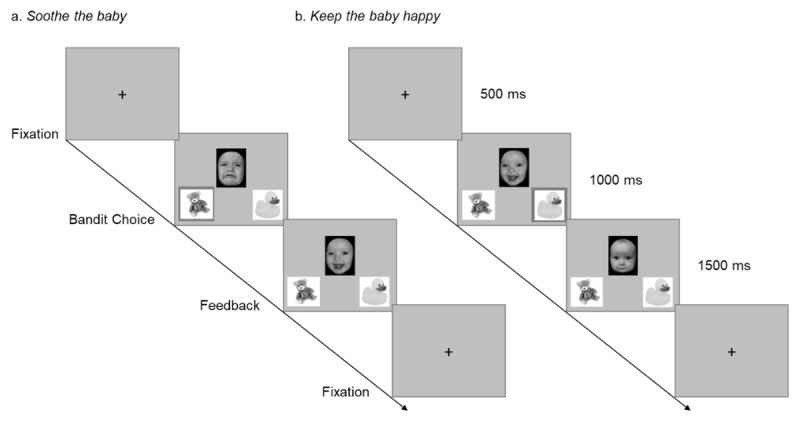
Maternally adapted version of a two-arms bandit task. *Note*. Timeline of events within a trial. Feedback can be a sad, neutral or happy baby face. **a)** “Soothe the baby” condition (with a positive outcome); **b)** “Keep the baby happy” condition (with a neutral outcome).

We developed two maternally adapted versions of a two-armed bandit task (Gittins, 1979; Whittle, 1980) in Psychopy 3.0 coder (Peirce et al., 2019) (Figure 2), specifically designed to simulate a playful caregiving interaction with an infant. These versions differed only in the baseline emotional context presented to participants (crying vs happy infant). The task was specifically structured to allow for the modelling of learning and decision-making processes using reinforcement learning frameworks. In particular, participant choices were modelled to estimate individual learning rates (how rapidly feedback updated value expectations), decision stochasticity (the degree of exploration versus exploitation), and subjective valuation of neutral outcomes. The tasks were initially piloted in an independent sample of 30 participants (see Appendix 1 and Figures S1–S2).

In Study 1, we tested the validity of the task and explored cognitive modelling strategies that were subsequently applied in Study 2. Briefly, participants were asked to select one of two bandits (i.e., toys) on each trial. Following selection, they were presented with the outcome (photos of real crying, neutral, and happy baby faces). Each bandit had an underlying probability of displaying one of the three outcomes, which switched every 40 trials. The only difference between the two experimental conditions was the baseline emotional state of the baby at the start of each trial. Before and after the task participants were presented with a rating task (Appendix 2.1) where they had to rate from 0–100 the emotional valence of the stimuli used and the hypothesised ‘temperament’ of the infant.

Further details on the hypotheses defined prior to data collection and analysis are reported in Appendix 2.1 and are also available online (Costantini, 2023).

### Procedure

In Study 1, participants were randomly allocated to one of the two tasks by drawing from an opaque container a piece of paper with an anonymous subject ID (obtained from a random-number generator software (https://www.randomizer.org/) and a condition number:

1. “Soothe the baby”: on each trial participants were shown a crying baby face and their task was to use one of the two bandits to soothe the baby (Figure 2a).
2. “Keep the baby happy”: on each trial participants were shown a smiling baby face and their task was to use one of the two bandits to keep the baby happy (Figure 2b).

In each trial, participants were asked to select one of the two bandits (i.e., teddy bear toy or duck toy) and were then provided with one of: positive feedback; negative feedback; or neutral feedback. The probabilities of these outcomes varied according to the underlying reward structure (Figure S3), such that the bandit that was most beneficial changed over time. The bandits remained in the same spatial location on every trial, but their location was randomised between participants.

Before and after the bandit task, participants were asked to rate the emotions and temperaments of the baby faces on a scale from 0 to 100 (sad to happy and difficult to easy temperament, respectively). This was done to test how negative, neutral, and positive the stimuli were perceived before starting the task and to check whether performing the task changed their values.

In Study 2, we selected only the *“Soothe the baby”* condition to characterise learning and decision-making processes in pregnant women. This decision was based on findings from Study 1 showing greater variability in learning rates in this condition, enhancing the opportunity to detect associations with mental health measures.

Data from participants’ choices and feedback during the task were then modelled using reinforcement learning frameworks to extract individual learning and decision-making parameters for subsequent analyses.

### Analyses

#### Model specification

All analyses were performed using R version 4.2.2 (R Core Team, 2021) and models were fitted in Stan (Stan Development Team., n.d.), using the rstan interface in R. We fitted two models to our data. The models are described conceptually here; mathematical details are given in Appendix 2.4–2.5. Reward feedback is numerical and the mapping between value and emotion was: –1 = negative (crying baby); 0 = neutral; 1 = positive (happy baby). After each choice, the participant receives social feedback and uses this feedback to update the value of the toy they chose. In both models, we used the Rescorla-Wagner rule (Rescorla and Wagner, 1972) to update the value estimates. These value estimates then guide choice through a Softmax decision rule (Sutton & Barto, 2018). The learning rule was characterised by a learning rate, α; the Softmax choice rule was characterised by a noise parameter that controls the stochasticity in choice (inverse temperature τ). Briefly, the learning rate α refers to how quickly the participants update their knowledge about the best bandit after receiving feedback on trial *t*. The inverse temperature τ parameter indicates the exploration-exploitation trade-off in the decision-making process. Values close to 0 represent elevated levels of stochasticity or randomness (i.e., greater exploration), and higher levels represent lower levels of stochasticity (i.e., greater exploitation). We refer to this basic model as the ‘delta rule’ model.

It is entirely plausible that the psychological experience of the social outcomes differs from their nominal value. For instance, the neutral outcome may not be experienced as neutral—indeed, we hypothesise that it will depend on the baseline emotional context. That is, a neutral outcome may be experienced as positive or negative depending on one’s starting or *reference* point (Tversky & Kahneman, 1979). Moreover, the experience of sad or happy may not be equidistant from the “neutral” outcome. Therefore, a second model included a reference point, η, that represents the emotional intensity in the stimulus that is experienced as neutral. Including this reference point allows for a non-linear mapping between nominal outcome and psychological value or utility (Figure S4). We will refer to this model as the ‘reference point’ model. We used hierarchical Bayesian modelling to a) identify which of these two models provides a better account of the observed choice behaviour, and b) estimate posterior distributions of the parameters of interest from the winning model. The exact hierarchical specification is given in Appendix and illustrated in Figure S5.

#### Model fitting and inference

Both models were fitted using four chains and 11,500 iterations per chain, where the first 1,500 samples were discarded as ‘burn-in’ (where the sampler has not yet “found” the region in the parameter space where most of the posterior density is located). Several model diagnostic checks were performed (Appendix 2.7). After assessing these model diagnostics, we retained every 10^th^ sample to reduce autocorrelation (thinning).

Once a model was fitted, we extracted its marginal log-likelihood using bridge sampling (Gronau et al., 2017) (‘bridgesampling’ package (Gronau et al., 2020) for R), which automatically penalises more complex or flexible models. The ratio of the marginal likelihoods of the two models is the Bayes Factor (Lee & Wagenmakers, 2014), which specifies how much the evidence (i.e., the data) should shift our (posterior) belief in one model over the other. Our inferences are based on the “winning” model, according to the Bayes Factor estimate (Jeffreys, 1998; Wetzels & Wagenmakers, 2012).

To identify effects of the baseline emotional context, model parameters depended linearly on the task condition. The coefficients associated with task condition then directly reflected the effect size on the parameter(s). We computed the 95% Highest-Density Intervals (HDI) (Kruschke, 2018) of the posterior distributions of the coefficient of the task condition factor (i.e., “Soothe the baby” vs “Keep the baby happy”) and reported the posterior probability of this parameter being greater or smaller than 0. We used Bayesian linear regression models to explore the relation between the task condition on the difference between pre- and post-task ratings of emotions and temperament of the infant stimuli. We utilised the Brm function (*brms* package (Bürkner, 2017)) to perform these analyses.

To explore whether mental health problems associated with learning and decision-making processes, we employed separate regression models for each outcome (i.e., individual-level posterior median α, τ and η) for all our five main independent variables (i.e., depressive symptoms, trait and state anxiety, impulsivity, and personality difficulties), using weakly informative priors. Every model included the following covariates: age and task condition. The task condition was not included in Study 2 as participants completed only one condition. The regression coefficient is a population-level posterior median regression weight which refers to the average estimated effect size (regression coefficient) across the population, derived from a Bayesian hierarchical (or multilevel) model. Further details are provided in the Appendix 2.6.

In Study 1, we included both females and males to maximise statistical power. Because Study 2 was restricted to pregnant female participants, we also re-ran all models by limiting our student sample (Study 1) to female participants only (N = 162) to aid comparability.

In Study 2, we used the EPDS to evaluate depressive symptoms, which has been described having three factors indicating anhedonia, anxiety, and depressive symptoms (Paul & Pearson, 2020). Because anxiety and depressive (and anhedonia) symptoms may associate differentially with parameters such as learning rates, we dropped the anxiety items (items: 3–6) from the scoring of the EPDS and re-ran the analyses using this score.

## Results

### Demographics

Basic demographic characteristic of the participants included in the analyses for Study 1 and 2 are reported in Table 2. In Study 1, the mean (SD) age was 20, and the majority (84%) identified as female. In Study 2, the mean (SD) age was 29 years (SD = 1.51), with all participants being female. Among participants in Study 2, 62% completed the task during their first pregnancy, and 38% during a subsequent pregnancy.

**Table 2:** Demographic characteristics of participants included in the analyses in Study 1 (nulliparous female and male student participants) and Study 2 (ALSPAC pregnant women).


DEMOGRAPHICS	STUDY 1: STUDENT		STUDY 2: ALSPAC	
		
VARIABLES	N	MEAN (SD)/%	N	MEAN (SD)/%

Age	191	19.82 (2.73)	109	29.11 (1.51)

**Gender/Sex**				

Male	28	14.66%	0	0%

Female	161	84.29%	109	100%

Other	2	1.04%	–	–

**Education**				

A Level or Higher	180	94.2%	39	43.33%

O Level	–	–	37	41.11%

<O Level	–	–	14	15.56%

High-school diploma (=GCSE)	11	5.8%	–	–

**Performance task**				

In person	191	100%	25	22.9%

Online	0	0%	84	77.1%

**MENTAL HEALTH MEASURES**	**N**	**MEDIAN (IQR)**	**N**	**MEDIAN (IQR)**

**Depression score**				

SMFQ	190	7 (4–10)	–	–

EPDS	–	–	80	13 (9.5–15)

**Anxiety**				

STAI-I	190	38 (32–46)	–	–

STAI-II	190	46 (39–53)	–	–

**Personality difficulties**				

SAPAS	190	3 (2–4)	91	2 (1–3)

BIS-11	190	64 (58–69)	29	51 (49–59)


*Note*. IQR: Inter-quartile Intervals; ALSPAC: Avon Longitudinal Study of Parents and Children; SAPAS: Standardized Assessment of Personality Abbreviated Scale; MFQ: Mood and Feeling Questionnaire; EPDS: Edinburgh Post-Natal Depressive Scale; STAI: State-Trait Anxiety Inventory; BIS: Barrett Impulsivity Scale.

### Model fit and diagnostic

We found strong evidence favouring the reference point model over the delta rule model in both Study 1 (Bayes Factor, BF_ref,delta_ = 2.65e+46) and Study 2 (BF_ref,delta_ = 2.89e+31). Therefore, all inferences are based on the reference point model. All the model diagnostics were adequate in both studies (Figures S6, S7, S10, and S11).

### The effect of baseline emotional context (Study 1)

**Figure 3:**
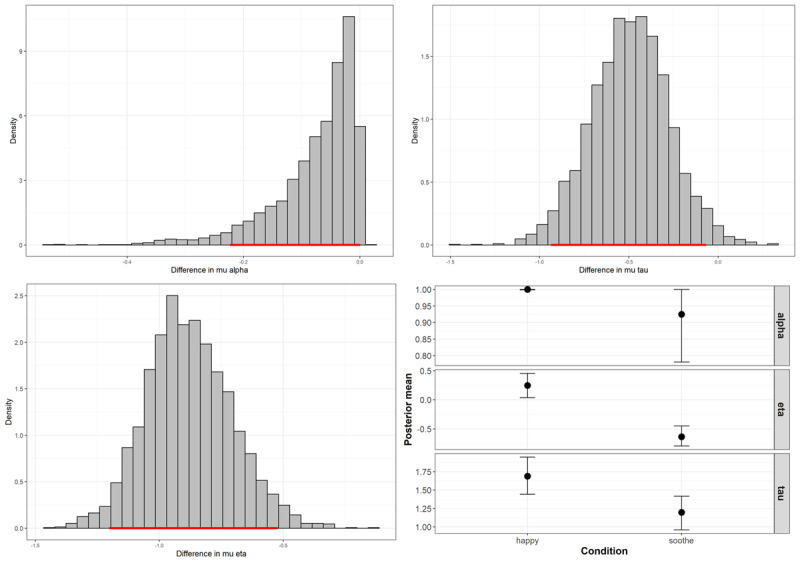
Effect of the baseline emotional state on the parameters of the reference model (Study 1). *Note*. The top two graphs and the bottom left graph illustrate the posterior differences for the population level means between the two conditions. The 95% highest density intervals are marked with the horizontal red line at the base of the histogram bars. The bottom right graph shows the median and 95% credible (highest density) intervals of the posterior densities for the population level mean learning rate, reference point, and inverse temperature.

Analysis of the 95% highest density intervals (HDIs) indicated strong evidence for an effect of the baseline emotional context on both the reference point and inverse temperature parameters, and some evidence for an effect on the learning rate parameter (Figure 3).

Participants randomised to the “Keep the baby happy” condition had higher reference point values (population-level posterior median = 0.25, 95% HDI: 0.03 to 0.45) compared to those in the “Soothe the baby” condition (population-level posterior median = –0.64, HDI: –0.80 to –0.45), indicating that participants in the “Keep the baby happy” condition evaluated the neutral outcome as more negative relative to participants in the “Soothe the baby” condition, who tended to perceive the neutral outcome more positively.

Participants in the “Keep the baby happy” condition also showed higher inverse temperature values (population-level posterior median = 1.69, HDI: 1.44 to 1.94) relative to those in the “Soothe the baby” condition (population-level posterior median = 1.20, HDI: 0.99 to 1.44), indicating a greater tendency toward deterministic choice behaviour.

Learning rates were higher and less variable in the “Keep the baby happy” condition (population-level posterior median = 1.00, HDI: 0.99 to 1.00) compared to the “Soothe the baby” condition (population-level posterior median = 0.92, HDI: 0.77 to 1.00), where greater individual variability was observed.

### Ratings of facial expressions and temperament

Participants in Study 1 and Study 2 rated infant facial expressions of emotion and temperament before and after the bandit task. In both studies, the ratings showed that the three facial expressions were distinguishable on both scales. In Study 1, on the emotion rating scale, the neutral expression was positioned approximately equidistant between sad and happy expressions, supporting the validity of the stimuli (Figure S3). The context manipulation did not have an effect on most post-task ratings. However, for temperament ratings, there was some evidence that the “Keep the baby happy” group rated the happy face more positively than the “Soothe the baby” group (mean difference = 4.39, 95% CrI: 0.14 to 8.78) (Table 3). In Study 2, participants also distinguished the three expressions (Figure S4), and there were no changes in emotion or temperament ratings following the bandit task (Table 3).

**Table 3:** This table illustrates whether the context manipulation affected the post-test rating scores on both the emotions’ and temperament’ ratings.


RATING EMOTION	N	POSTERIOR MEAN DIFFERENCE (95% CrIs)	Rhat

Negative	203	0.02 (–2.84 to 2.85)	1

Neutral	203	–0.88 (–3.55 to 1.72)	1

Happy	203	0.39 (–1.67 to 2.49)	1

**RATING TEMPERAMENT**	**N**	**POSTERIOR MEAN DIFFERENCE (95% CrIs)**	**Rhat**

Negative	203	0.86 (–4.49 to 6.44)	1

Neutral	203	–1.41 (–6.49 to 3.53)	1

Happy	203	4.39 (0.14 to 8.78)	1


*Note*. The table reports brms estimates on the final rating score on both emotions and temperament with the condition. The reference condition is the “Soothe the baby”. 95% CrIs: Credible Intervals. Rhat is a convergence diagnostic statistic used in Markov Chain Monte Carlo (MCMC) models. An Rhat value below 1.05 is generally considered a strong indication that the chains have converged well.

### The association between mental health measures and model parameters

#### Study 1

The associations between depressive symptoms, anxiety measures, impulsivity, and learning and decision-making parameters were generally small, with most 95% credible intervals (CrI) including zero (Table 4).

**Table 4:** This table reports the posterior median effect size and 95% CrI representing the association between levels of personality difficulties, depression, anxiety and impulsivity and the parameters of interest in both Study 1 and Study 2.


MENTAL HEALTH MEASURE^1^	PARAMETERS^2^	STUDY 1 (NULLIPAROUS PARTICIPANTS)				STUDY 2 (ALSPAC PARTICIPANTS)			
	
		NUMBER OF PARTICIPANTS	ESTIMATE (MEDIAN POSTERIOR DISTRIBUTION)	95% CRI	RHAT	NUMBER OF PARTICIPANTS	ESTIMATE (MEDIAN POSTERIOR DISTRIBUTION)	95% CRI	RHAT

	**Alpha**								

Personality difficulties (SAPAS)		190	–0.08	–0.20 to 0.04	1.00	91	–0.18	–0.30 to –0.04	1.00

Depressive symptoms (MFQ in Study 1 and EPDS in Study 2)		190	–0.06	–0.17 to 0.06	1.00	80	–0.14	–0.30 to 0.01	1.00

Depressive symptoms (EPDS without anxiety items)			–	–	–	80	–0.14	–0.30 to 0.03	1.00

State anxiety (STAI-Y1)		190	–0.03	–0.13 to 0.08	1.00	–	–	–	–

Trait anxiety (STAI-Y2)		190	–0.06	–0.18 to 0.05	1.00	–	–	–	–

Impulsivity (BIS-11)		190	–0.08	–0.19 to 0.03	1.00	–	–	–	–

	**Tau**								

Personality difficulties (SAPAS)		190	–0.03	–0.08 to 0.02	1.00	91	0.01	–0.08 to 0.10	1.00

Depressive symptoms (MFQ in Study 1 and EPDS in Study 2)		190	0.00	–0.07 to 0.08	1.00	80	–0.07	–0.15 to 0.02	1.00

Depressive symptoms (EPDS without anxiety items)		–	–	–	–	80	–0.06	–0.15 to 0.02	1.00

State anxiety (STAI-Y1)		190	–0.05	–0.10 to –0.00	1.00	–	–	–	–

Trait anxiety (STAI-Y2)		190	–0.00	–0.05 to 0.05	1.00	–	–	–	–

Impulsivity (BIS-11)		190	–0.02	–0.08 to 0.02	1.00	–	–	–	–

	**Eta**								

Personality difficulties (SAPAS)		190	0.07	–0.01 to 0.14	1.00	91	–0.02	–0.12 to 0.07	1.00

Depressive symptoms (MFQ in Study 1 and EPDS in Study 2)		190	–0.00	–0.06 to 0.05	1.00	80	0.02	–0.07 to 0.11	1.00

Depressive symptoms (EPDS without anxiety items)		–	–	–	–	80	0.01	–0.08 to 0.10	1.00

State anxiety (STAI-Y1)		190	0.04	–0.03 to 0.11	1.00	–	–	–	–

Trait anxiety (STAI-Y2)		190	0.01	–0.06 to 0.08	1.00	–	–	–	–

Impulsivity (BIS-11)		190	–0.07	–0.14 to –0.01	1.00	–	–	–	–


*Note*. CrI: Credible Intervals; ALSPAC: Avon Longitudinal Study of Parents And Children; SAPAS: Standardized Assessment of Personality Abbreviated Scale; MFQ: Mood and Feeling Questionnaire; EPDS: Edinburgh Post-Natal Depressive Scale; STAI: State-Trait Anxiety Inventory; BIS: Barrett Impulsivity Scale. Rhat is a convergence diagnostic statistic used in Markov Chain Monte Carlo (MCMC) models. An Rhat value below 1.05 is generally considered a strong indication that the chains have converged well.

For depressive symptoms, there was weak evidence of an association with the learning rate (population-level posterior median regression weight (i.e., coefficient) = –0.06, 95% CrI: –0.17 to 0.06), and no evidence for associations with the inverse temperature (coefficient = 0.00, 95% CrI: –0.07 to 0.08) or the reference point (coefficient = –0.00, 95% CrI: –0.06 to 0.05).

For state anxiety, there was moderate evidence for a negative association with inverse temperature (coefficient = –0.05, 95% CrI: –0.10 to –0.00), suggesting that higher levels of state anxiety were associated with more exploratory decision-making behaviour. No strong evidence was observed for associations between state anxiety and either learning rate (coefficient = –0.03, 95% CrI: –0.13 to 0.08) or reference point (coefficient = 0.04, 95% CrI: –0.03 to 0.11). There was no evidence for associations between trait anxiety and any model parameters.

We found little evidence that higher impulsivity was negatively associated with lower learning rates (coefficient = –0.08, 95% CrI: –0.19 to 0.03) and reference point (coefficient = –0.07, 95% CrI: –0.14 to –0.01), but not with inverse temperature (coefficient = –0.02, 95% CrI: –0.08 to 0.02) (Table 4).

#### Study 2

In Study 2, we found moderate evidence that participants with higher personality difficulties had lower learning rates (coefficient = –0.18, 95% CrI: –0.30 to –0.04). There was no evidence for associations between personality difficulties and either inverse temperature (coefficient = 0.01, 95% CrI: –0.08 to 0.10) or reference point values (coefficient = –0.02, 95% CrI: –0.12 to 0.07).

We also found some suggestive evidence indicating that higher symptoms of depression in pregnancy were associated with a lower learning rate (coefficient = –0.14, 95%CrI: –0.30 to 0.01) and more randomness in decision-making processes (coefficient = –0.07,95%CrI: –0.15 to 0.02).

Repeating the analyses after excluding the anxiety-related items from the EPDS yielded similar estimates for all parameters (Table 4).

Restricting the Study 1 sample to female participants (N = 162) produced results consistent with those of the full sample. Effect sizes for associations between mental health measures and model parameters were slightly larger in the female-only sample (Appendix 2.7), although the overall pattern of findings remained the same.

## Discussion

To our knowledge, this is the first study to (i) have developed a learning-based task specifically designed to simulate an interaction with an infant and analysed data using advanced Bayesian modelling techniques (Study 1 and 2), (ii) evaluated the role of infant’s baseline emotional context on learning and decision-making processes (Study 1), and (iii) explored whether mental health difficulties associated with parameters of interest in both males and nulliparous females (Study 1) and pregnant women (Study 2).

In Study 1, we found strong evidence that the baseline infant emotional state affected the *utility* of the neutral feedback and decision-making processes, making explorative strategies more likely during an interaction with a crying baby as compared to when interacting with a happy baby. These results agree with our hypothesis that reinforcement learning processes are sensitive to contextual effects (Bavard et al., 2018; Vandendriessche et al., 2021). Taken together, these findings suggest that the ‘baseline emotional state’ of the infant (i.e., a potential proxy for their temperament) affects learning and decision-making processes in nulliparous participants and it may affect parents’ behaviours (even though this was not explicitly tested in our studies).

Participants with greater personality difficulties exhibited slower learning in both Study 1 and Study 2. The direction of the effect was consistent across studies, though the evidence was stronger in Study 2. Given the overlap in credible intervals and the absence of an a priori interaction hypothesis, this difference may reflect random variation and should be interpreted with caution. These findings align with impaired learning mechanisms reported in the literature about personality difficulties (Henco et al., 2020) and are especially noteworthy given the existing evidence showing that maternal personality difficulties have important effects on offspring development (Eyden et al., 2016). Recent epidemiological evidence has suggested that women with greater personality difficulties may be exhibiting fewer sensitive behaviours when interacting with their infant (Høivik et al., 2018; Nath et al., 2020). One possible explanation is that reduced flexibility in belief updating may contribute to less sensitive responses to infant cues, which could be targeted by interventions.

In Study 2, we also found evidence suggesting that higher antenatal depressive symptoms were associated with lower learning rates. These findings align with recent systematic review and meta-analysis (Halahakoon et al., 2020) showing that depressed participants were more likely than healthy controls to show a small impairment in reward-related learning processes. However, these studies focused on a clinical population, limiting generalisability to population-level mental health difficulties, and excluded studies using social stimuli as feedback.

This study should be evaluated in the context of several limitations. First, absence of a control condition (e.g., monetary outcomes) limits our ability to determine the specificity of observed associations to social feedback. Second, while we adjusted for age (Xia et al., 2020), we were unable to account for other potential confounders, such as trauma or PTSD (Jowett et al., 2020), and neurodevelopmental disorders (Hedley et al., 2018; Sethi et al., 2018), making these estimates purely correlational. Third, potential selection biases due to differential attrition in Study 2 (i.e., ALSPAC) (Lawlor et al., 2019) may limit the generalisability of our findings to the general population of pregnant women. Moreover, although data collection in Study 2 was timed to coincide with the second trimester—when social cognitive changes have been reported (Cárdenas et al., 2020)—the absence of longitudinal data (e.g., pre-, during-, and post-pregnancy) and negative controls (e.g., expectant fathers) precludes attributing observed effects specifically to pregnancy-related biological or psychological processes.

To conclude, the baseline context, representing different infant’s emotional states, influenced learning and decision-making parameters. This could potentially indicate the relevance of including the child in parent-infant interventions as their temperament may be an important effect modifier of parental learning and decision-making behaviours. Further, antenatal depressive symptoms and greater personality difficulties were associated with slower learning from a social infant stimulus. Disrupted learning and decision-making processes may be one of the salient mechanisms underpinning disrupted parent-infant interaction when poor mental health is present, and thus these processes could be implicated in the intergenerational transmission of poor maternal mental health to offspring risk.

## Data Accessibility Statement

All analysis code will be made available on OSF in time for publication (10.17605/OSF.IO/QV6UN). This code should allow other researchers to reproduce the task, the modelling, and the analyses for Study 1. Anonymised data for Study 1 have also been made available. Stimuli used in the task have also been made available.

ALSPAC data access is through a system of managed open access. The steps below highlight how to apply for access to the data included in the data note and all other ALSPAC data:

1. Please read the ALSPAC access policy (http://www.bristol.ac.uk/media-library/sites/alspac/documents/researchers/data-access/ALSPAC_Access_Policy.pdf) which describes the process of accessing the data and samples in detail, and outlines the costs associated with doing so.
2. You may also find it useful to browse our fully searchable research proposals database (https://proposals.epi.bristol.ac.uk/?q=proposalSummaries), which lists all research projects that have been approved since April 2011.
3. Please submit your research proposal (https://proposals.epi.bristol.ac.uk/) for consideration by the ALSPAC Executive Committee. You will receive a response within 10 working days to advise you whether your proposal has been approved.

## Additional File

The additional file for this article can be found as follows:

10.5334/cpsy.134.s1 Appendix. Supplementary materials including pilot study data, task reward structure, detailed methods, hierarchical Bayesian model specifications, sensitivity analyses, and additional diagnostic figures and tables.

## Footnotes

All independent variables were normalised using z scores to have mean of 0 and SD of 1.

Higher scores of alpha indicate faster updating strategies (i.e., higher learning rate); Lower scores of eta indicate that the neutral stimulus was perceived more as a gain, whilst higher (i.e., positive) scores of eta indicate that the neutral stimulus was perceived as a loss. Higher scores of tau indicate more exploitative/deterministic decision-making strategies, whilst lower scores indicate more explorative/random decision-making strategies.
