# Supplementary material for: Investigating Learning, Decision-Making, and Mental Health in Pregnancy: Insights From a UK Cohort Study
Source: Comput Psychiatr. 2025 Sep 3;9(1):142–58. doi: 10.5334/cpsy.134 (PMC12427613; doi:10.5334/cpsy.134)
Supplement: Appendix. — Supplementary materials including pilot study data, task reward structure, detailed methods, hierarchical Bayesian model specifications, sensitivity analyses, and additional diagnostic figures and tables. [file cpsy-9-1-134-s1.pdf]

Investigating Learning, Decision-Making, and Mental Health in Pregnancy: Insights from a UK Cohort Study

Ilaria Costantini[1,2], Axel Montout[3], Paul Moran[1,4,5], Daphne Kounali[4], Rebecca M. Pearson[1,6,7,8], Casimir J.H. Ludwig[2]

[1]Centre for Academic Mental Health at the University of Bristol, Oakfield House, Bristol, United Kingdom.

[2]School of Psychological Science, University of Bristol, Bristol, United Kingdom.

[3]Bristol Veterinary School, University of Bristol, Bristol, United Kingdom.

[4]NIHR Biomedical Research Centre at the University Hospitals Bristol NHS Foundation Trust, Bristol, GB

[5]Population Health Sciences, Bristol Medical School, University of Bristol, Bristol, United Kingdom.

[6]Bristol NIHR Biomedical Research Centre, Bristol, UK

[7]MRC Integrative Epidemiology Unit, University of Bristol, Oakfield House, Oakfield Grove, Bristol, UK

1 Pilot Study

We conducted a pilot study to explore whether we were able to estimate meaningful parameters and to derive weakly informative priors for Study 1 and Study 2 from the obtained posterior distributions. We also used the Pilot data to explore different accuracy thresholds we then applied in Study 1 and Study 2.

1.1 Participants

30 participants, mean age (33.1 years), 60% females, 40% males, were recruited into the study as a convenience sample and were randomly allocated to the “Soothe the baby” or “Keep the baby happy” conditions (n=15 per condition).

1.2 Task

The task was the same as that used in Study 1 and Study 2, however, the reward structure differed slightly (see below for details on the reward structure).

T = 200: total number of trials.

S = 4: number of discrete switches in probability; 50 number of consecutive trials in which the probabilities remain constant.

Pi, neutral = 0.2: probability of neutral outcome.

Pi, sad ∈ {0.3, 0.5}: probability of sad outcome.

Pi, happy ∈ {0.3, 0.5}: probability of happy outcome.

1.3 Simulating performance employing the following reward structure

Figure S1. Reward landscape and reward structure employed in the Pilot study (n=30).

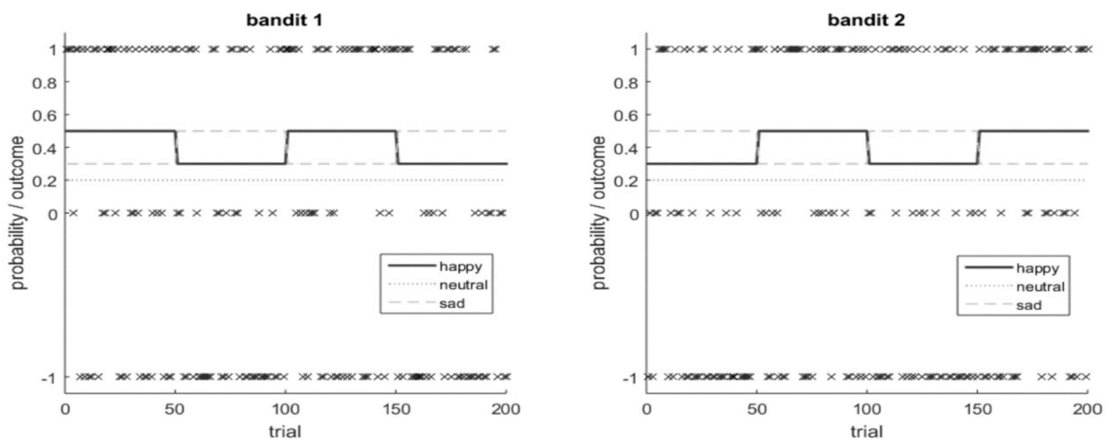

Note. SFigure 1 illustrates the underlying reward structure of the task. In this task there are two bandits (toys): X_1 and X_2. Choosing a bandit can result in three outcomes: sad, neutral, or happy baby face. These are represented numerically as -1,0,1. Each bandit X_i has a multinomial reward distribution with event probabilities p_{ij}, where $\sum_{j=1}^{3} p_{ij} = 1$. We set $p_{i,neutral} = 0.20$ throughout the experiment. In other words, both bandits are equally likely to give a neutral outcome and this probability remains constant. The other two probabilities mirror each other: $p_{1,sad}(t) = p_{2,happy}(t)$ and $p_{1,happy}(t) = p_{2,sad}(t)$. The probability of sad and happy outcomes swaps over for a given bandit periodically, so that $p_{i,sad}(t) = p_{i,happy}(t + n)$, where n is the number of trials in a "block" in which the probabilities remain stationary. For a given window of trials, w, we ensure that the frequency of outcomes on offer for a given bandit matches the nominal probability underlying that temporal window.

1.4 Findings

The two reinforcement learning models were fit using the same procedures as in the main study (but with less informed priors). Posterior distributions obtained in this Pilot study were then used to inform the group-level priors in our main studies (specified below in section 2.5 below). For the sake of brevity, detailed results from the Pilot study are not reported.

Figure S2. Bandit choices of two participants when completing the "Soothe the baby" condition and the “Keep the Baby Happy” condition.

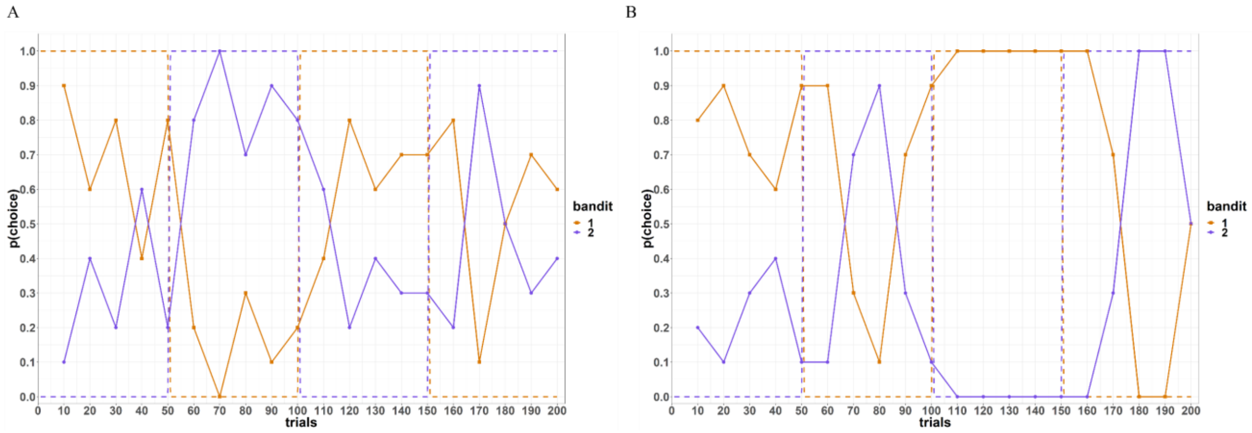

Note. SFigure 2A, “Soothe the baby” condition; SFigure 2B, “Keep the Baby Happy” condition. The coloured dotted lines indicate the which bandit was better across the different trial windows. The dots and continuous lines in the graph show the participants’ choices averaged over a window of ten trials. Both participants adapted to the switches in outcome probabilities, although even within an epoch in which the outcome probabilities were constant, they sometimes switched their preferences (e.g. panel A, trials 150-200).

2 Methods and Materials: Study 1 and Study 2

2.1 Pre-specified hypotheses

Regarding the development of a computational model to apply to learning and decision-making processes in expectant women, we fitted two models based on a Rescorla-Wagner rule (Rescorla and Wagner, 1972) to update the value estimates (i.e., learning rate, α parameter) and a Softmax decision rule (Sutton & Barto, 2018) to guide their decision-making processes (inverse temperature parameter τ). The basic model is a simple delta-rule model that contains just these two parameters for any one participant. An augmented model additionally allowed for the utility of the neutral outcome to vary. In this model, we introduced a third parameter, η, which we refer to as the 'reference point'. We hypothesised that the model where the utility of the neutral outcome was allowed to vary would best fit the data in this Study, but also in Study 2 (i.e., pregnant women). The models are described in more detail in Appendix 2.3.

To examine the impact of the baseline emotional context on participants' feedback evaluation and learning and decision-making strategies, we hypothesised that the reference point η and the inverse temperature τ parameters would differ across conditions. Specifically, we hypothesised that the neutral outcome would be experienced as more positive in the "Soothe the baby" condition where the participants always start the interaction with a baby in a crying/sad baseline state, compared to the "Keep the baby happy" condition, where the baseline state of the baby is happy. In addition, we hypothesised that the baseline emotional context would affect the decision-making strategies (inverse temperature parameter τ), so that participants completing the "Soothe the baby" condition would show more "explorative" strategies than those completing the "Keep the baby happy" condition. The rationale behind this hypothesis is that in this condition the child baseline emotional state is already a negative one (i.e., distressed crying face), and participants may be more willing to explore their options to obtain a positive outcome as they "have nothing to lose".

To evaluate whether symptoms of depression, anxiety, and personality difficulties associated with learning and decision-making processes, we hypothesised that participants with higher scores on anxiety measures would have higher learning rates α, driven by a heightened attention toward negative feedback(Aylward et al., 2019). Conversely, participants with higher scores on measures of depressive symptoms and personality difficulties would be slower in re-appraising the new best bandit and would experience greater difficulties in updating their strategies, resulting in a lower learning rate (Mukherjee et al., 2020). We hypothesise two slightly different mechanisms here: 1) individuals with high depressive symptoms would either not revise their strategy or would be slower to do this (because they are less sensitive to the rewarding effect of the positive feedback) and, conversely, 2) participants with high scores on dysfunctional personality traits would have slower learning rates because of an impaired cognitive flexibility.

2.2 Measures of mental health problems

In Study 1, we obtained the following measures:

The State-Trait Anxiety Inventory (STAI-Form Y1 and Y2) is a 40-item self-report measure that was used to assess state and trait anxiety (Spielberger, 1983). Cronbach alpha was 0.93 for both state and trait anxiety items, indicating optimal internal validity.

Personality difficulties were measured using the 8-items Standardised Assessment of Personality–Abbreviated Scale (SAPAS) (Moran et al., 2003). Higher scores indicated a higher risk of being diagnosed with a personality disorder. Cronbach alpha was 0.47, which is consistent with that reported in other studies (Germans et al., 2012).

Depressive symptoms were assessed using the 13-item Short-Mood and Feeling Questionnaire (SMFQ) (Messer et al., 1995). Higher scores indicate more depressive symptoms. Cronbach alpha was 0.86, indicating optimal internal validity.

Impulsivity was assessed using the 30-item Barratt Impulsiveness Scale 11 (BIS-11) (Barratt, 2007). Higher scores indicated more impulsive behaviours. Cronbach alpha was 0.80, indicating optimal internal validity.

In Study 2:

The psychological measures considered in ALSPAC-G1 and ALSPAC-G1 partners pregnant women were as follows:

Depressive symptoms were assessed with the Edinburgh Postnatal Depression Scale (EPDS)(Murray & Cox, 1990) (*n*=80, 73% of total sample). The EPDS is a validated 10-item self-report measure designed to screen women for depression both during and after pregnancy. In this study we used continuous symptom scores to maximise statistical power. Cronbach's alpha was 0.72 indicating acceptable internal consistency.

Personality difficulties were assessed using the Standardised Assessment of Personality - Abbreviated Scale (SAPAS)(Moran et al., 2003) (*n*=91, 83% of the total sample). The SAPAS is a validated 8-item screening interview. Cronbach's alpha was 0.58 indicating an acceptable internal consistency.

Despite BIS-11 was collected in this sample, it was not included in the analyses because there was more than 70% missingness and we did not have previous measures to use as auxiliary variables for imputation.

2.3 Rating of facial expressions

Participants were asked to rate the emotion (from sad to happy, with middle values indicating neutral) and the temperament (from difficult to easy) of the infant faces employed in the baby bandit task, before and after the completion of it. Values ranged from 0-100 and participants were free to select any value clicking on a slide before the image of the infant face which was placed at the centre of the screen. Each facial expression was coded one per time (both for the emotional and temperament rating) and the order of rating was randomised. Instructions about the rating procedure were provided both at the start and at the end of the task and a reminder “Rate the emotion!”/ “Rate the temperament!” was always present above the stimuli. Findings are discussed in the main text and are depicted in the below figures (S3 and S4).

Figure S 3. Rating of emotions and temperament of the three baby facial expressions used in the bandit task, pre- and post- completion of the task, by condition (Study 1).

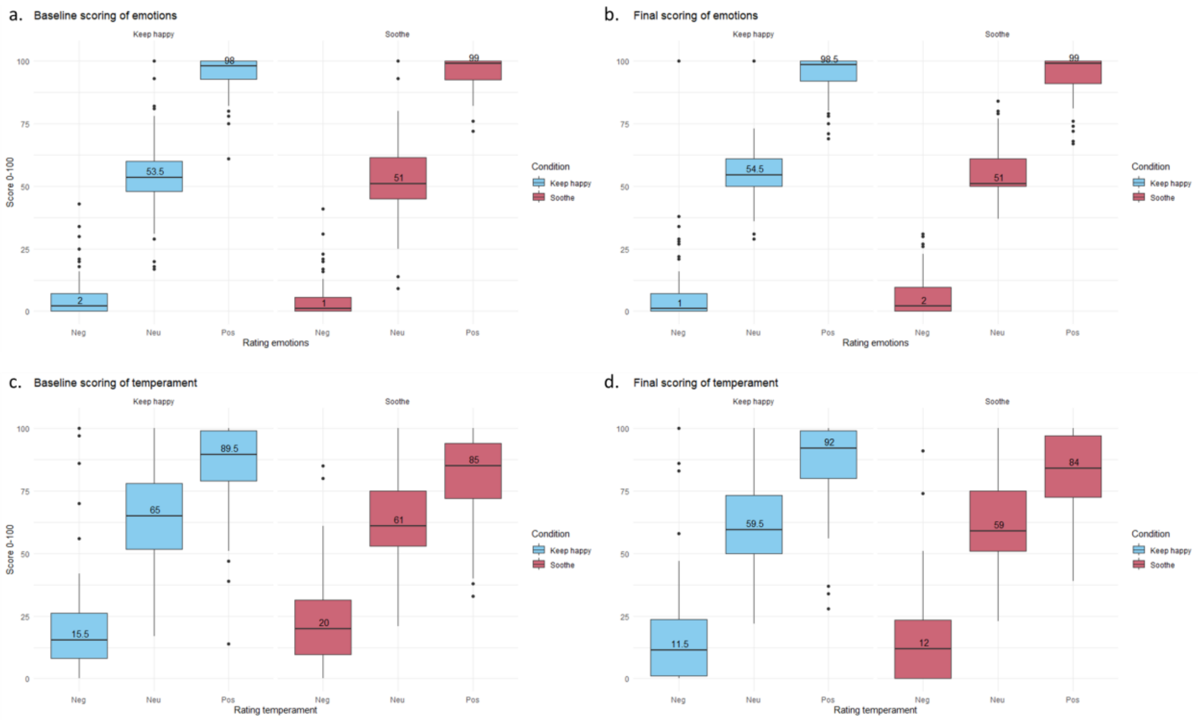

Note. Box plots of the ratings pre- and post- task for both the emotions and the temperament of the baby depicted in our stimuli. Section a. illustrates the median and inter-quartile range of emotion rating of the infant across the three baby facial expressions (i.e., negative, neutral, and positive) before starting the bandit task. Section b. reports the emotional scoring at the end of the task.

Sections c. and d. illustrate the ratings of temperament (i.e., from very difficult, 0, to very easy, 100) across the three baby facial expressions, pre- and post- completion of the bandit task, respectively.

Figure S 4. Rating of emotions and temperament of the three baby facial expressions used in the bandit task, pre- and post- completion of the task (Study 2).

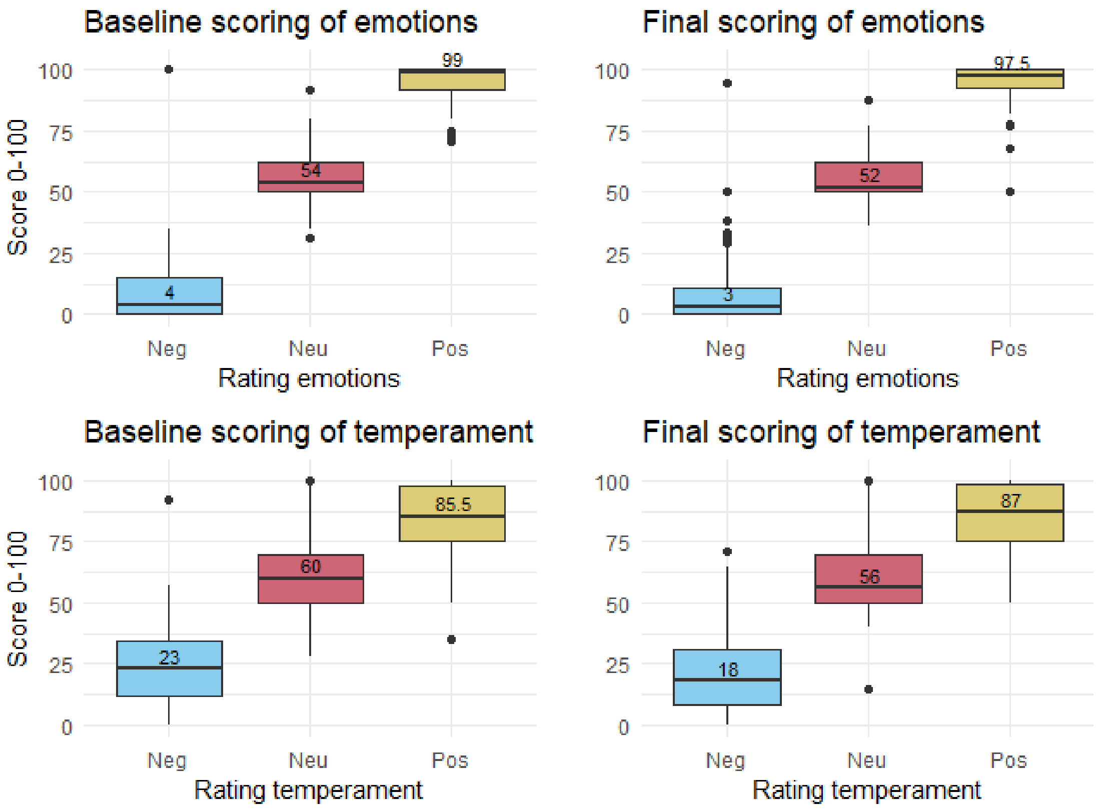

Note. ALSPAC participants completed only one version of the bandit task (i.e., "Soothe the baby"). Conventions and lay-out as in Figure 4.

2.4 Bandit task

The task was developed and presented using the Psychopy 3.0 coder on PC computers. In this task there are two bandits (toys): X_1 and X_2. Choosing a bandit can result in three outcomes: sad, neutral, or happy baby face. These are represented numerically as $-1,0,1$. Each bandit X_i has its own multinomial reward distribution with event probabilities p_{ij}, where $\sum_{j=1}^{3} p_{ij} = 1$. We set $p_{i,neutral} = 0.15$ throughout the experiment. In other words, both bandits are equally likely to give a neutral outcome and this probability remains constant. The other two probabilities mirror each other: $p_{1,sad}(t) = p_{2,happy}(t)$ and $p_{1,happy}(t) = p_{2,sad}(t)$. The probability of sad and happy outcomes swaps over for a given bandit periodically, so that $p_{i,sad}(t) = p_{i,happy}(t + n)$, where n is the number of trials in a "block" in which the probabilities remain stationary. For a given window of trials, w, we ensure that the frequency of outcomes on offer for a given bandit matches the nominal probability underlying that temporal window. In summary, we have the following task parameters that defined the reward structure (Figure S5):

$\mathrm{T} = 200$: total number of trials.

$\mathrm{s} = 5$: number of discrete switches in probability.

$\mathrm{n} = \mathrm{T}/\mathrm{s} = 40$: number of consecutive trials in which the probabilities remain constant.

$p_{i,neutral} = 0.15$: probability of a neutral outcome.

$p_{i,sad} \in \{0.25,0.6\}$: probability of a sad outcome.

$p_{i,happy} \in \{0.25,0.6\}$: probability of a happy outcome.

$\mathrm{w} = 40$: window of consecutive trials over which the frequency of outcomes matches the nominal probabilities.

Figure S 5. Reward structure of maternally adapted task in Study one and Study two.

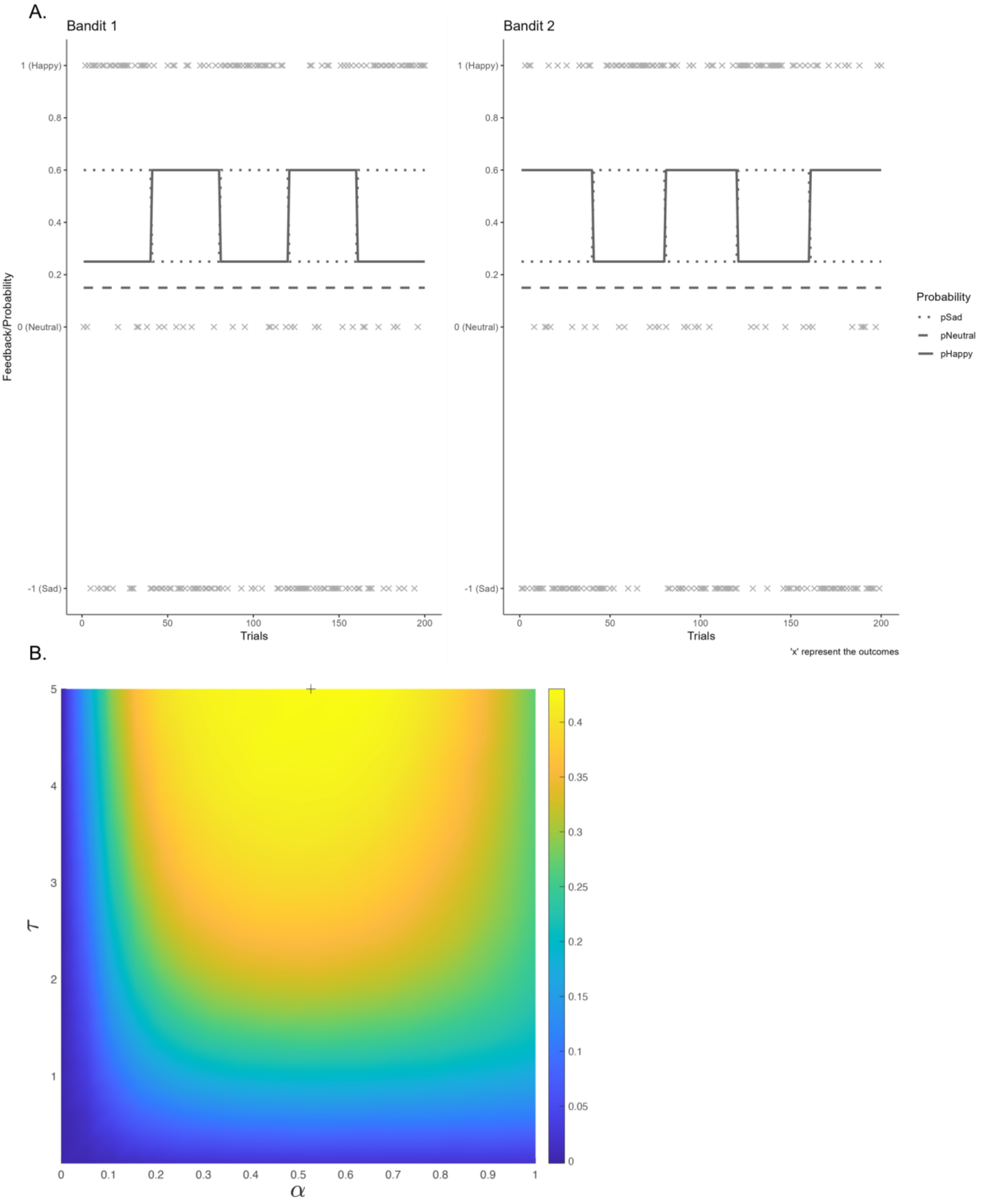

Note. **Figure S5a** illustrates the underlying reward structure of the task. In this task there are two bandits (toys): X_1 and X_2. Choosing a bandit can result in three outcomes: sad, neutral, or happy baby face. In our task, there were $n = 200$ trials to complete and the nominal probability for the best bandit switched every $w = 40$ trials. **Figure S5b** illustrates the reward landscape obtained via simulations of a simple delta rule model performed in MATLAB Version: 9.13.0 (R2022b)(The

MathWorks Inc., 2022). The aim of the simulation was to evaluate whether the reward structure itself would push participants towards extreme learning rates, which might limit individual differences in the estimates for this parameter. The warmer the colour (yellow/orange) the better the performance at the task (i.e., in terms of accumulated reward). Optimal simulated value for alpha fell at 0.7 and a relatively high inverse temperature. Values had some spread, indicating a good performance could have been obtained with a range of values.

2.5 Hierarchical Bayesian Modelling

A hierarchical Bayesian model consists of specifying the probabilistic relations between stimuli, model parameters, and observed data.

In the bandit task, we have a time series of binary choices and (trinary) outcomes following those choices. In this section, we first specify the reference point model at the individual level, recognising that the simpler delta rule model is nested by virtue of setting the reference point $\eta = 0$. We then specify the full probabilistic model that encompasses the population and individual level parameters and their relation to the data. We use the learning rate parameter as an example, but the same logic applies to the inverse temperature and reference point parameters.

For a given individual, we assume their learning rate comes from a population level distribution. This population level distribution has a certain mean and variance. The hierarchical Bayesian model provides posterior densities for the population level mean and variance, as well as for each individual level learning rate. These posterior densities may then be used for inference. Data from both conditions were fit jointly by contrast coding the ‘soothe the baby’ and ‘keep the baby happy’ conditions as -0.5 and 0.5 respectively and letting the population level mean of the parameters be a linear function of this independent variable. In this way, the slope of this linear function directly indexes the difference in the population level means of the two conditions and we can assess the posterior probability of this difference

being greater or less than 0. It also allows us to compare the effect of the context manipulation between the different parameters directly on a common scale (Vandekerckhove et al., 2011).

Participant i performed $\mathrm{t} = 1 \ldots T$ trials in condition $z_i \in \{-0.5, 0.5\}$ (corresponding to the 'soothe' and 'keep happy' conditions, respectively). On trial $t = 1$, the participant assigns both bandits a utility of 0, makes a (random) choice $y(t)$ and observes the outcome $r(t)$. For subsequent trials then, the participant updates the value (or utility) assigned to the chosen bandit j as follows:

$v_j(t+1) = v_j(t) + \alpha_i\left[u_j(t) - v_j(t)\right]$, where

$u(t) = r(t)$ if $r(t) \in \{-1, 1\}$

$u(t) = \mathrm{r}(t) - \eta_i$ if $r(t) = 0$.

Parameters α and η, are the learning rate and reference point, respectively. Note that when $\eta = 0$, this model is equivalent to the basic delta-rule model. The term in the square brackets is the familiar prediction error: the difference between the experienced and expected utility of the outcome. If the prediction error is positive (experienced outcome is better than expected), the value assigned to bandit j on the next trial is increased; if the prediction error is negative (experienced outcome is worse than expected), the value is decreased. With the updated values in place, the participant makes a choice that is probabilistically related to the difference in value between the two bandits, using a SoftMax choice rule:

$$p_j(t+1) = \frac{\mathrm{e}^{\tau_i v_j(t+1)}}{\sum_{k=1}^{2} \mathrm{e}^{\tau_i v_k(t+1)}},$$

which gives the probability of choosing bandit j on trial $t + 1$. Parameter τ is the inverse temperature that controls the degree of randomness in the choice: as $\tau \rightarrow 0$, the choice is essentially random, but as τ increases the participant is more likely to choose the bandit with the maximum value.

The hierarchical Bayesian model is aimed at identifying a) the mean of the population level distributions from which the three individual level parameters of interest are drawn, and b) the individual level posterior distributions of these parameters. The former is of critical interest regarding our experimental manipulation of the context; the latter are of critical interest with regard to the individual differences analyses. In the following $\psi \in \{\alpha, \tau, \eta\}$, refers to a single parameter of interest. The hierarchical structure is specified as follows.

$\beta_{0\psi} \sim \mathcal{N}(\mu_0, \sigma_0)$, $\beta_{1\psi} = \mathcal{N}(\mu_1, \sigma_1)$ correspond to the intercept and slope of a linear model on the population level means:

$$\mu_\psi = \beta_{0\psi} + \beta_{1\psi} z$$

$$\sigma_\psi \sim \text{half-Cauchy}(0{,}0.2)$$

$$\widehat{\psi_i} \sim \mathcal{N}(\mu_\psi, \sigma_\psi)$$

$\psi_i = -l + \Phi(\hat{\psi}_i) \times h$, where

$$l = \begin{cases} 0 \text{ if } \psi \in \{\alpha, \tau\} \\ -1 \text{ if } \psi = \eta \end{cases} \text{ and } u = \begin{cases} 1 \text{ if } \psi = \alpha \\ 5 \text{ if } \psi = \tau \\ 2 \text{ if } \psi = \eta \end{cases}.$$

Here, the linear model specifies the mean of the population level normal distribution from which an individual level model parameter $\hat{\psi}_i$ is drawn. To improve sampling in Stan, the individual level parameter is initially specified on a linear, unbounded scale, and drawn from a Gaussian distribution. The normalised parameter is then passed through an inverse cumulative normal distribution, Φ and rescaled to the interval $[l, l+h]$. The same transformation can be applied for the population level means μ_ψ to put these on their appropriate, bounded scale.

For the population level regression parameters, we need to choose the hyperpriors. Note that a "standard" choice of $\mu_0 = \mu_1 = 0$ and $\sigma_0 = \sigma_1 = 1$ is inappropriate: these normal deviates are combined to form the population level mean parameters μ_ψ that, when

transformed to their bounded scale, peak near the extremes of the scale and have a broad dip in the middle. Therefore, we conducted an initial pilot study with 30 participants (15 in each condition) to estimate more appropriate priors (see Section S1 above). The pilot study suggested that learning rates below 0.5 were unlikely, and the posterior density of μ_α was left-skewed with a peak close to 1. In addition, for both μ_τ and μ_η, values near the lower and upper limits were extremely unlikely. We introduced a prior bias in favour of learning rates between 0.5 and 1, by setting $\mu_0 = 0.5$ for $\beta_{0\alpha}$. For all other $\beta_{0\psi}$ we set $\mu_0 = 0$. For all slope parameters $\beta_{1\psi}$, we set $\mu_1 = 0$. Note that the slope parameters directly correspond to a normalised measure of the effect of the starting state manipulation—by setting $\mu_1 = 0$ there was no prior bias in the direction of this effect size (even though we did have directional expectations, at least for μ_τ and μ_η). We ensured a drop-off in the prior densities toward the end points of the scale for all ψ by setting $\sigma_0 = \sigma_1 = \frac{1}{2}\sqrt{(2)}$. With these hyperparameters, the prior density for μ_α is left-skewed, with most of its mass (~75%) between 0.5 and 1. For μ_τ and μ_η, the prior densities peak at 2.5 and 0 respectively, have a relatively flat plateau around this value and then drop off symmetrically according to an “inverted U” shape.

2.6 Brms model

We used the "0 + Intercept" notation in the model formula to explicitly include the intercept as a fixed, population-level effect rather than as a default intercept. This approach allows the intercept to be modelled similarly to a slope, giving it a more direct interpretation as a predictor of the outcome variable. We used *set_priors* command of *brm* to define the prior distributions for the parameters included in our models. We modelled the dependent variables (medians of individual level posteriors) using a skewed normal distribution to accommodate the skewness of the outcome data, especially in the α parameter (Study 1). Because we used a skew_normal distribution for the outcome, an additional parameter accounting for the residual standard deviation (‘sigma’) needs to be specified. A half-Cauchy prior distribution was chosen for the sigma parameter. Because of the heavy tails of this distribution, half-

Cauchy prior distributions are generally defined as “weakly informative” because it allows data to dominate if there is strong likelihood of the data in that region (Gelman, 2006).

Figure S 6. Schematic illustration of the hierarchical structure of the Bayesian model used to estimate group and individual-level parameters (α, τ, and η) from the behavioural data (Y1,1...J; Y2,1...J; Yn,1...J) obtained from each participant.

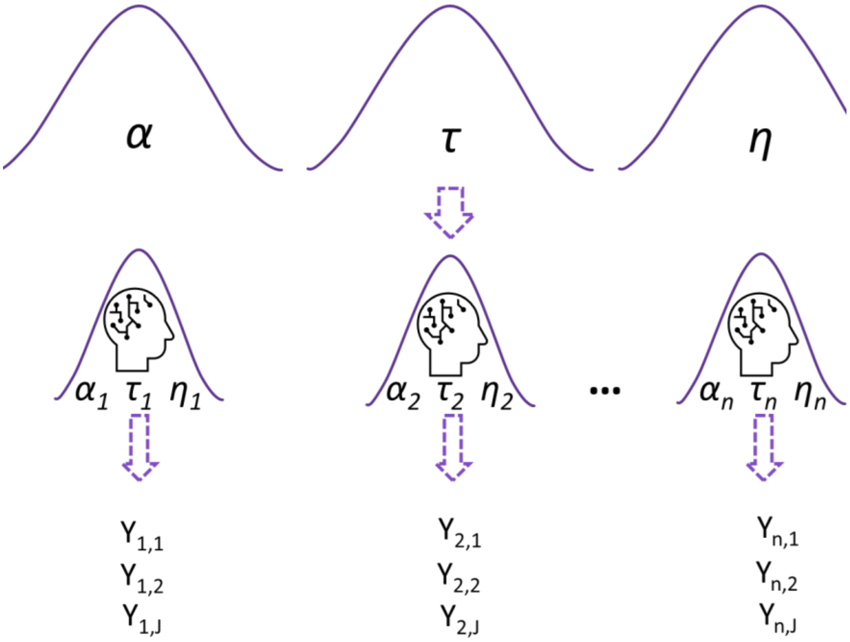

Note. The hierarchical Bayesian model assumes that individual-level parameters (α, τ, η) are drawn from population-level distributions, which are estimated from participants’ behavioural data. This structure allows simultaneous estimation of both group- and individual-level effects.

Figure S7. Behavioural data from three random subjects in Study 1 condition "Soothe the Baby".

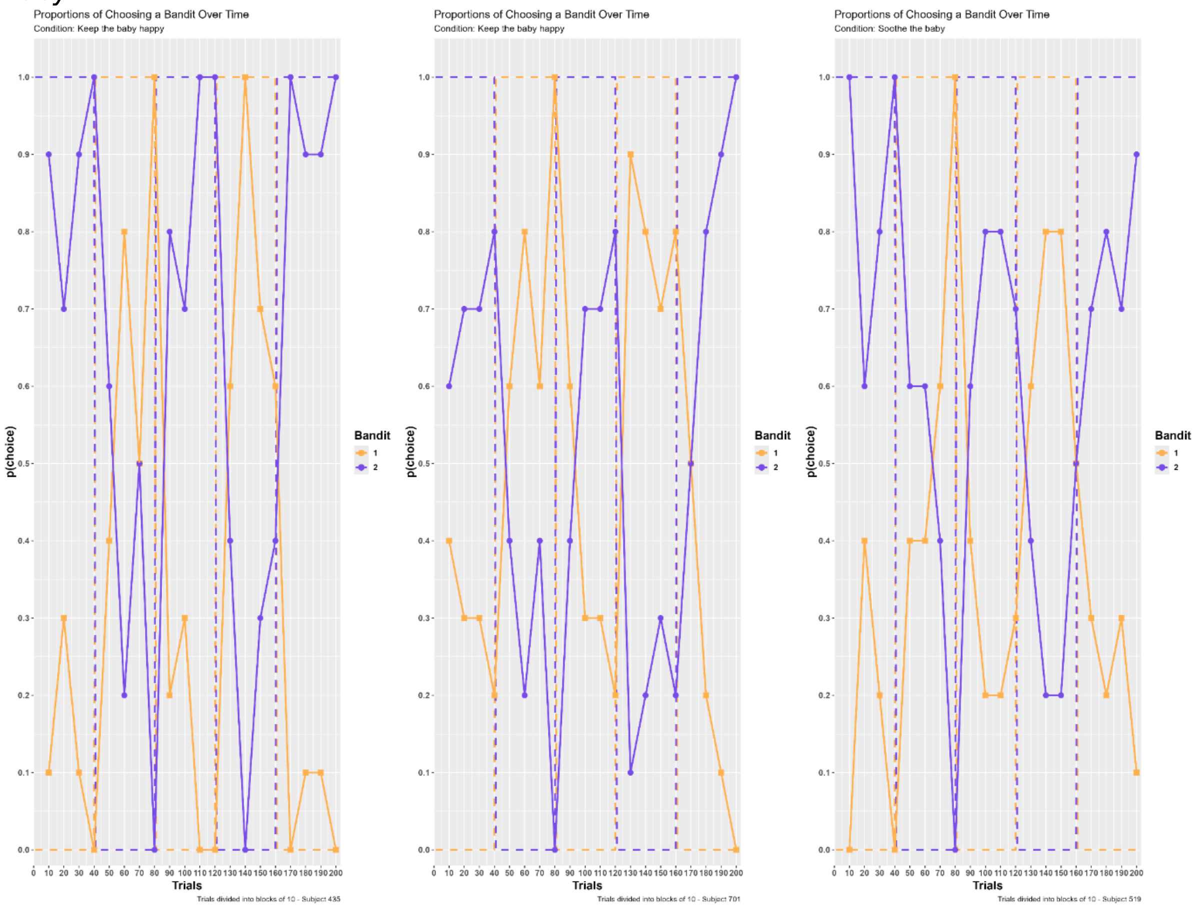

Note. These figures represent the proportion of actual choices made by three randomly selected subjects, shown every ten trials across the bandits. The dotted lines indicate which bandit was most likely to provide the most rewarding feedback (i.e., child smile) over 40 consecutive trials.

Figure S8. Behavioural data from three random subjects in Study 1 condition "Keep the Baby Happy".

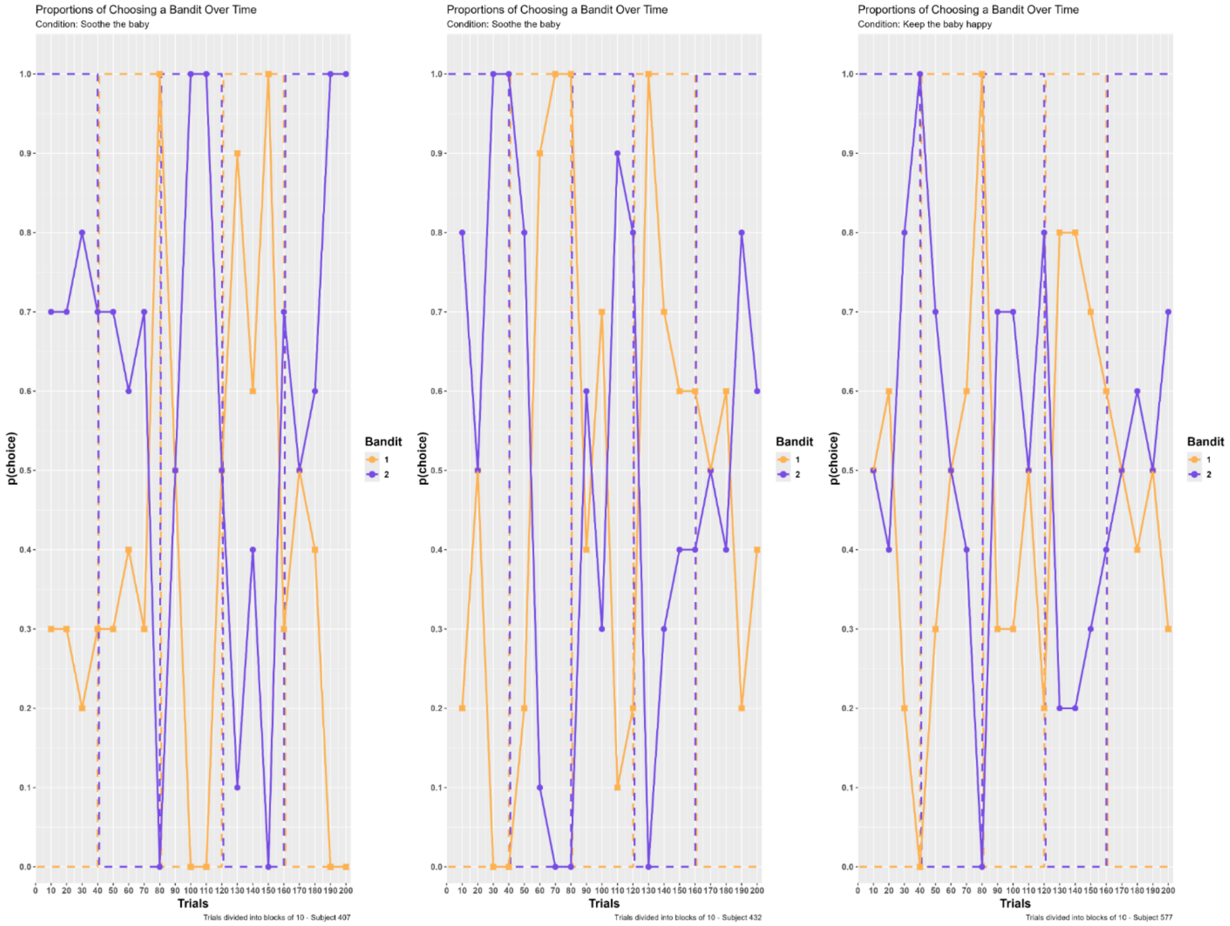

Note. These figures represent the proportion of actual choices made by three randomly selected subjects, shown every ten trials across the bandits. The dotted lines indicate which bandit was most likely to provide the most rewarding feedback (i.e., child smile) over 40 consecutive trials.

2.7 Model Diagnostics Study 1 and Study 2

The figures below report several model diagnostics that were performed in both studies to assess the convergence and covariance properties of the parameter estimates. Figure S8 and Figure S12 illustrate multiple useful MCMC diagnostics to investigate convergence across chains, in Study 1 and Study 2 respectively. Figures S8, S9, and S13 illustrate plots useful for the identification of collinearity between variables, depicting (Gelman et al., 2013)the effective sample size (n_{eff}) estimate. This estimate represents the number of independent draws from the posterior distribution of the estimand of interest, the larger the ratio n_{eff} to N is the better (Gelman et al., 2013).

Figure S10 illustrates the population-level posterior distributions of the estimated parameters in both Study 1 and Study 2.

Figures S11 and S14 represents the individual-level posterior distributions of the estimated parameters in Study 1 and Study 2, respectively.

Figure S9. Model ('reference point') diagnostics in Study 1 ('student sample').

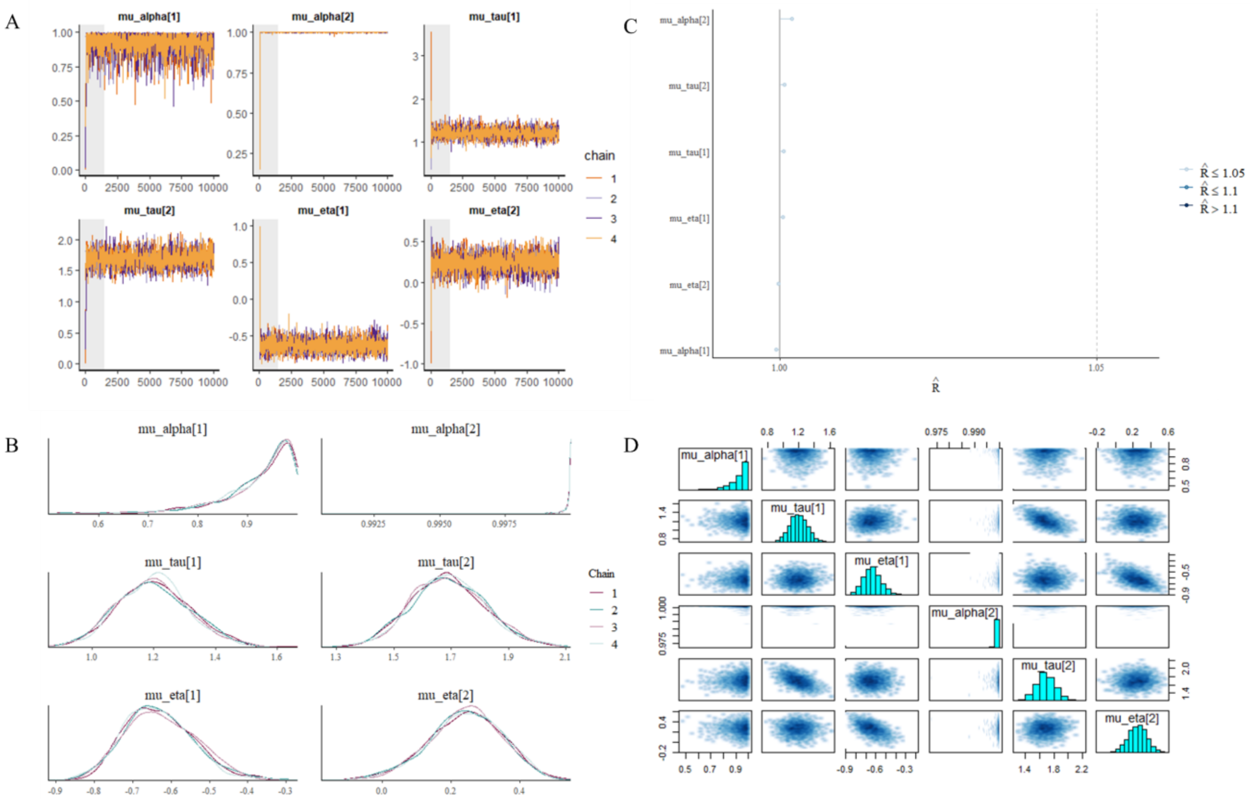

Note. Figure S8A ('caterpillar plot' or 'trace plot') is one of the multiple useful MCMC diagnostic visual tools to investigate convergence across chains. It illustrates the time series plots of Markov chains across all the iterations for all the parameters. The grey shaded area represents the *burn-in* period. The following part shows the 'post-warmup iterations. In addition, Figure S8B which plots overlaid density estimates also illustrates convergence and it is a useful tool to explore divergent chains, but it does not plot the chains across iterations. Figure S8C illustrates the Rhat values, which are all below 1.05, indicating good convergence and thus supporting reliability of the parameters estimated. Figure S8D ('banana plots') is useful to identifying collinearity between variables (which is characterised by narrow bivariate plots) and the presence of multiplicative non-identifiability (which are 'banana-shaped'). Here, we are interested in identifying collinearity or non-identifiability across parameters and not among the same parameter across conditions (which are necessarily correlated, given the linear model from which the population level mean parameters were derived). For example, the clear correlation mu_tau[1] and mu_tau[2] is expected and does not raise concerns about the model.

Figure S10. Model diagnostics ('reference point model') in Study 1 (student sample).

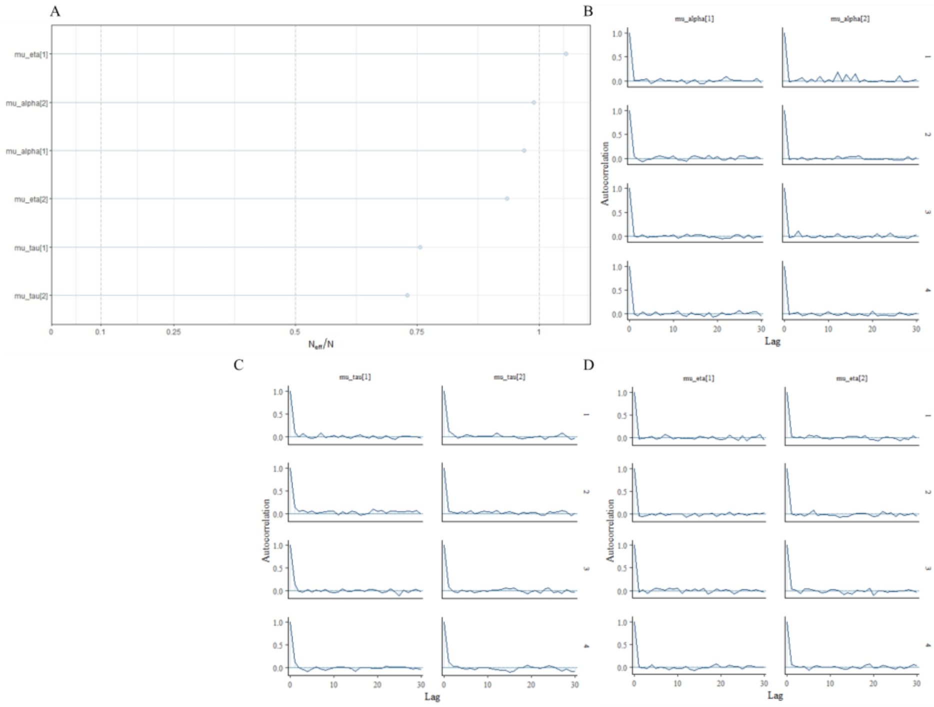

Note. Figure S11A ('mcmc_neff') depicts the n_{eff}/N ratio, the larger the ratio n_{eff} to N is the better (Gelman et al., 2013). The effective sample size (n_{eff}) estimate represents the number of independent draws from the posterior distribution of the estimand of interest. Because the draws from MCMC are not independent, it is important to consider the autocorrelation within the chains. For independent draws, the effective sample size is just the number of iterations. For correlated draws, the effective sample size will be lower than the number of iterations (Stan Development Team, 2022). Figures S11 B, C, and D further illustrates the degree of autocorrelation by chain and parameter after thinning by a factor of 10.

The population-level posterior mean distributions obtained in this sample (i.e., pregnant women) were comparable to those obtained in the student population when completing the same condition. For instance, the population-level posterior distribution of η was negative, thus indicating that the neutral stimulus was used as rewarding feedback to guide behaviour. However, the α parameter was more normally distributed than that in the student study, with the median of the population-level posterior mean distribution estimated at 0.74 (IQR: 0.71-0.77) (Figure S12).

Figure S11. Population-level posterior distributions of the estimated parameters in Study 1 ('student sample') and Study 2 ('ALSPAC sample').

A

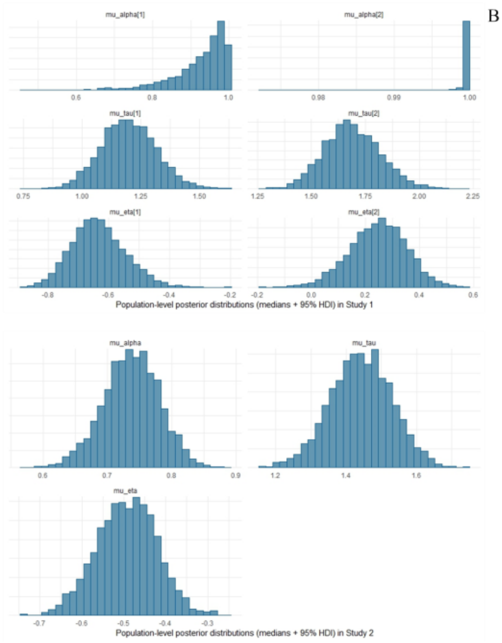

B

Parameter	Study 1	Study 2
Soothe the baby	***Median (IQR)***	***Median (IQR)***
**Alpha**	0.95 (0.89 to 0.98)	0.74 (0.71 to 0.77)
**Tau**	1.19 (1.12 to 1.43)	1.44 (1.38 to 1.50)
**Eta**	-0.64 (-0.70 to -0.58)	-0.49 (-0.44 to -0.36)
Keep the baby happy	***Median (IQR)***	***Median (IQR)***
**Alpha**	1 (1.00 to 1.00)	-
**Tau**	1.68 (1.60 to 1.78)	-
**Eta**	0.25 (0.18 to 0.32)	-

Note. Figures S12A illustrates the posterior distributions for the population level means in the analyses in Study 1 and Study 2 for the parameters: alpha, tau, and eta. Figure S12B reports the population-level posterior distributions' medians and IQR in Study 1 and Study 2 for all the estimated parameters.

Figure S12. Individual-level posterior distributions (medians + 95% HDI) among paraments in Study 1 ('student sample').

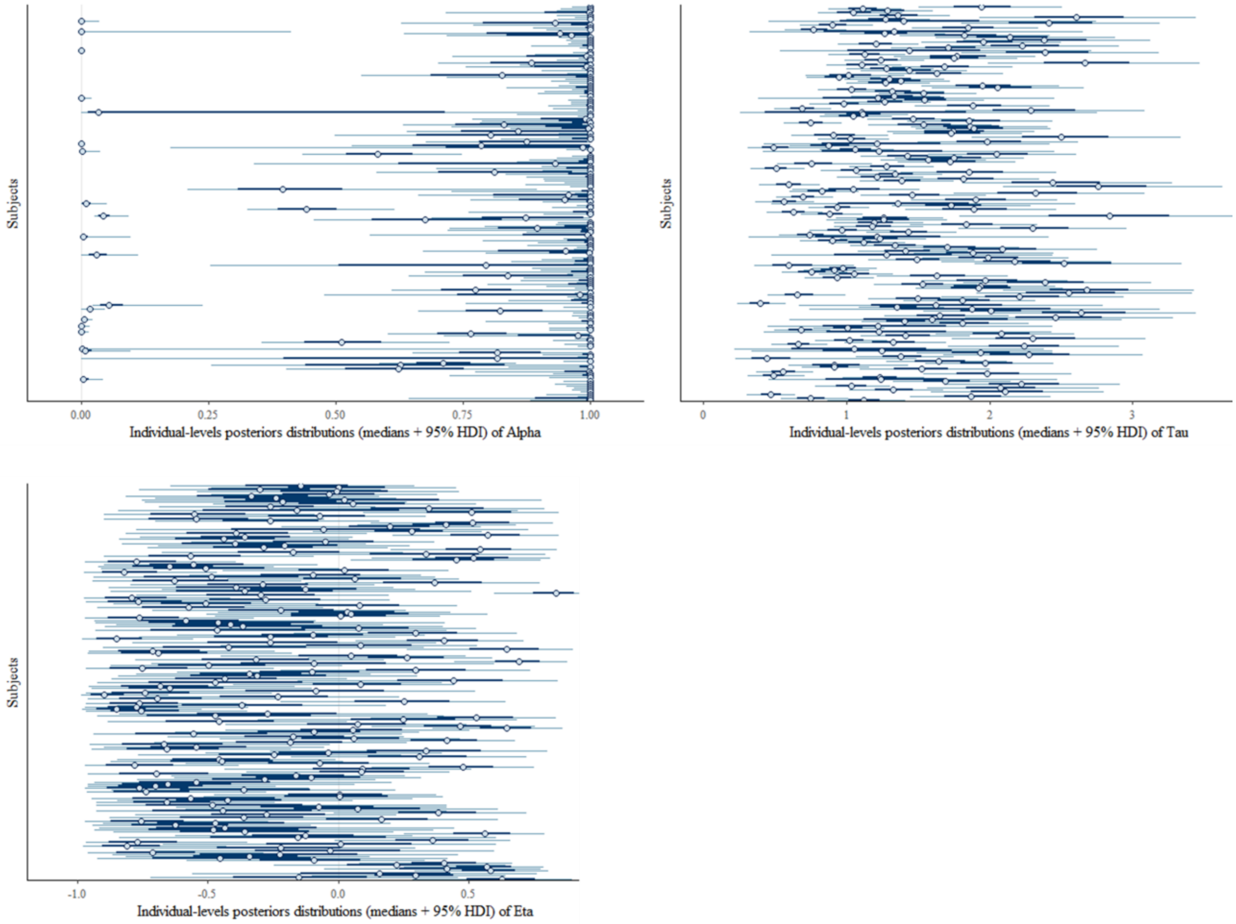

Note. Individual level medians and intervals of their posterior distribution for each parameters, showing 66% (darker colour) and 95% intervals.

Figure S13. Model diagnostics plots assessing convergency of the MCMC chains in Study 2 ('ALSPAC sample').

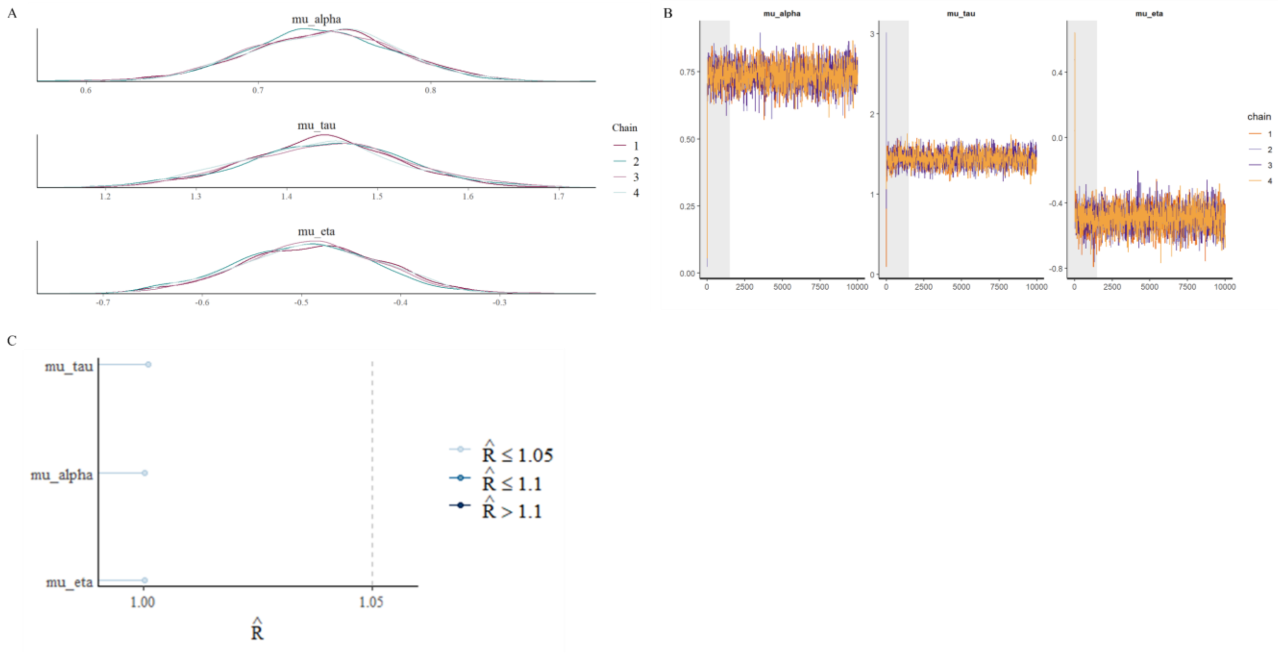

Note. Figure S14A plots overlaid density estimates also illustrates converges and it is a useful tool to explore divergent chains, but it does not plot each chain individually. In addition, Figure S14B ('caterpillar plot' or 'trace plot') is one of the multiple useful MCMC diagnostic visual tools to investigate convergence across chains. It illustrates the time series plots of Markov chains across all the iterations for all the parameters. The grey shaded area represents the *burn-in* period. The following part shows the 'post-warmup' iterations. Figure S14C illustrates the Rhat values (colour coded), which are all below 1.05 indicating good convergence and thus supporting reliability of the parameters estimated.

Figure S14. Model diagnostics plots assessing autocorrelation within chains in Study 2 ('ALSPAC sample').

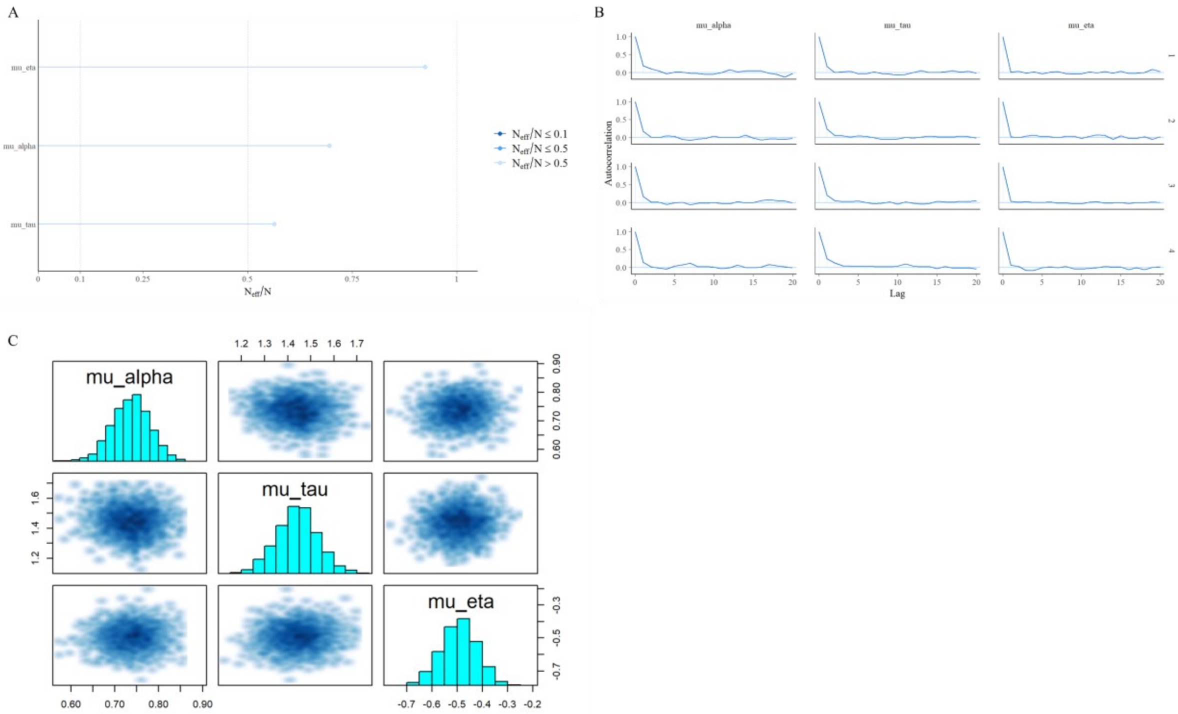

Note. Figures S15 A, B, and C represent the same diagnostic tests used described in the legend of Figures S11.

Figure S15. Individual-level posterior distributions (medians + 95% HDI) among paraments in Study 2 (ALSPAC participants).

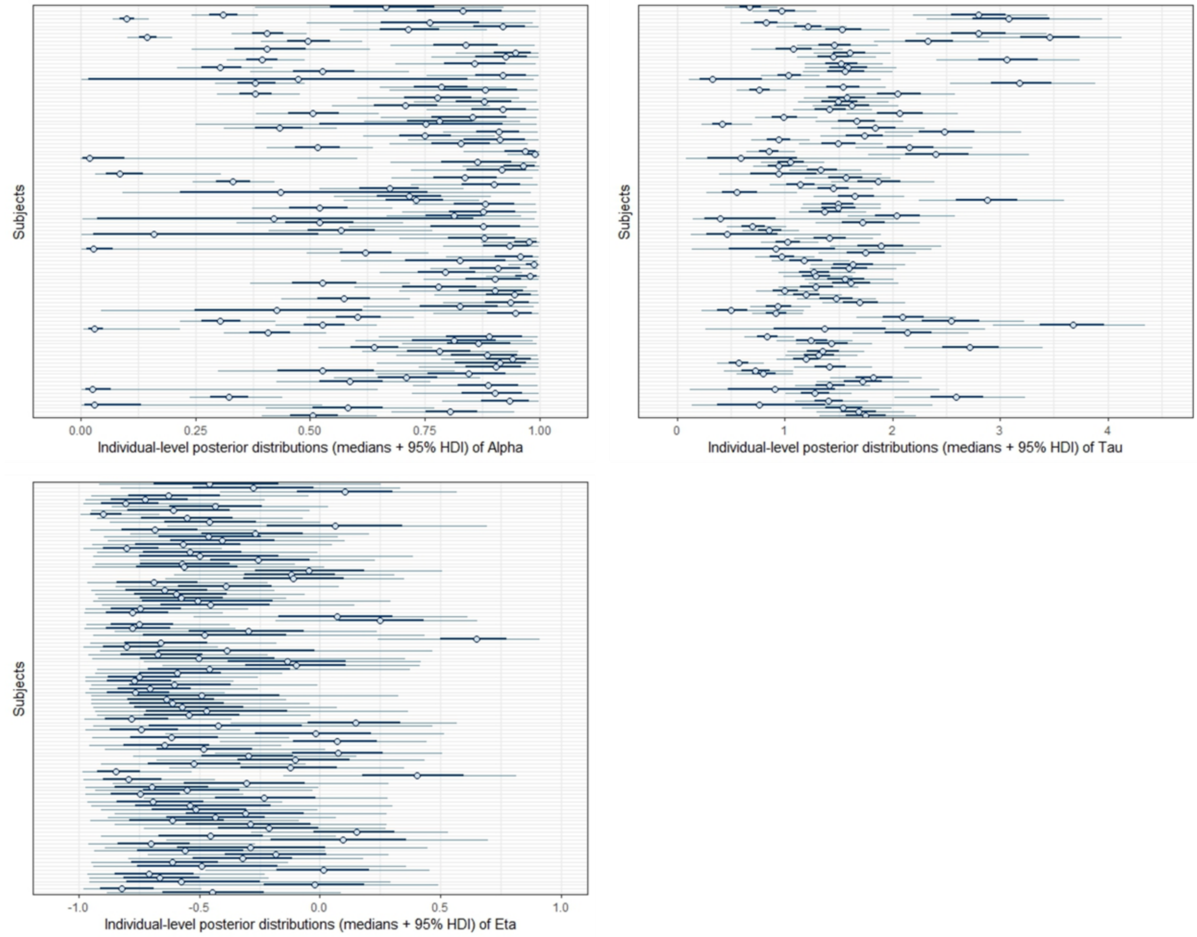

Note. Individual level medians and intervals of their posterior distribution for each parameters, showing 66% (darker colour) and 95% intervals.

2.8 Sensitivity Analysis

2.8.1 Model diagnostic and parameter estimates in Study 1’s female only sample

As sensitivity analysis, we restricted the model fitting and the analyses to female only participants (N=162). All model diagnostics were adequate as depicted in SFigures 17-19 indicating that reliable inferences could be made about this model too.

Figure S 16 Model ('reference point') diagnostics in Study 1 ('student sample') but restricting to females only participants.

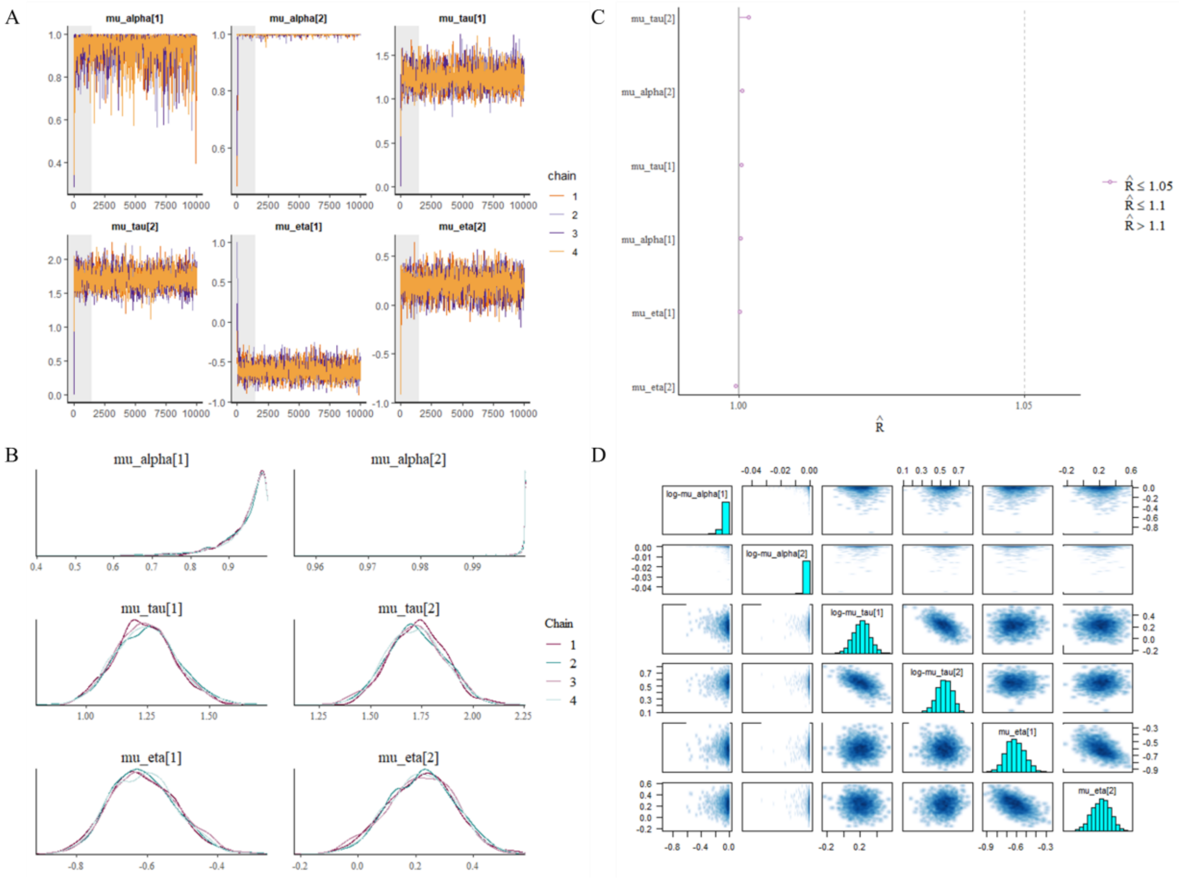

Note. Figure S17A (‘caterpillar plot’ or ‘trace plot’) is one of the multiple useful MCMC diagnostic visual tools to investigate convergence across chains. It illustrates the time series plots of Markov chains across all the iterations for all the parameters. The grey shaded area represents the burn-in period. The following part shows the ‘post-warmup iterations. In addition, Figure S17B which plots overlaid density estimates also illustrates convergence and it is a useful tool to explore divergent chains, but it does not plot the chains across iterations. Figure S17C illustrates by colour the Rhat values, which are all below 1.05, indicating good convergence and thus supporting reliability of the parameters estimated. Figure S17D (‘banana plots’) is useful to identifying collinearity between variables (which is characterised by narrow bivariate plots) and the presence of multiplicative non-

identifiability (which are ‘banana-shaped’). Here, we are interested in identifying collinearity or non-identifiability across parameters and not among the same parameter across conditions (which are necessarily correlated, given the linear model from which the population level mean parameters were derived). For example, the clear correlation mu_tau[1] and mu_tau[2] is expected and does not raise concerns about the model.

Figure S 17. Model diagnostics ('reference point model') in Study 1 (student sample) - females only.

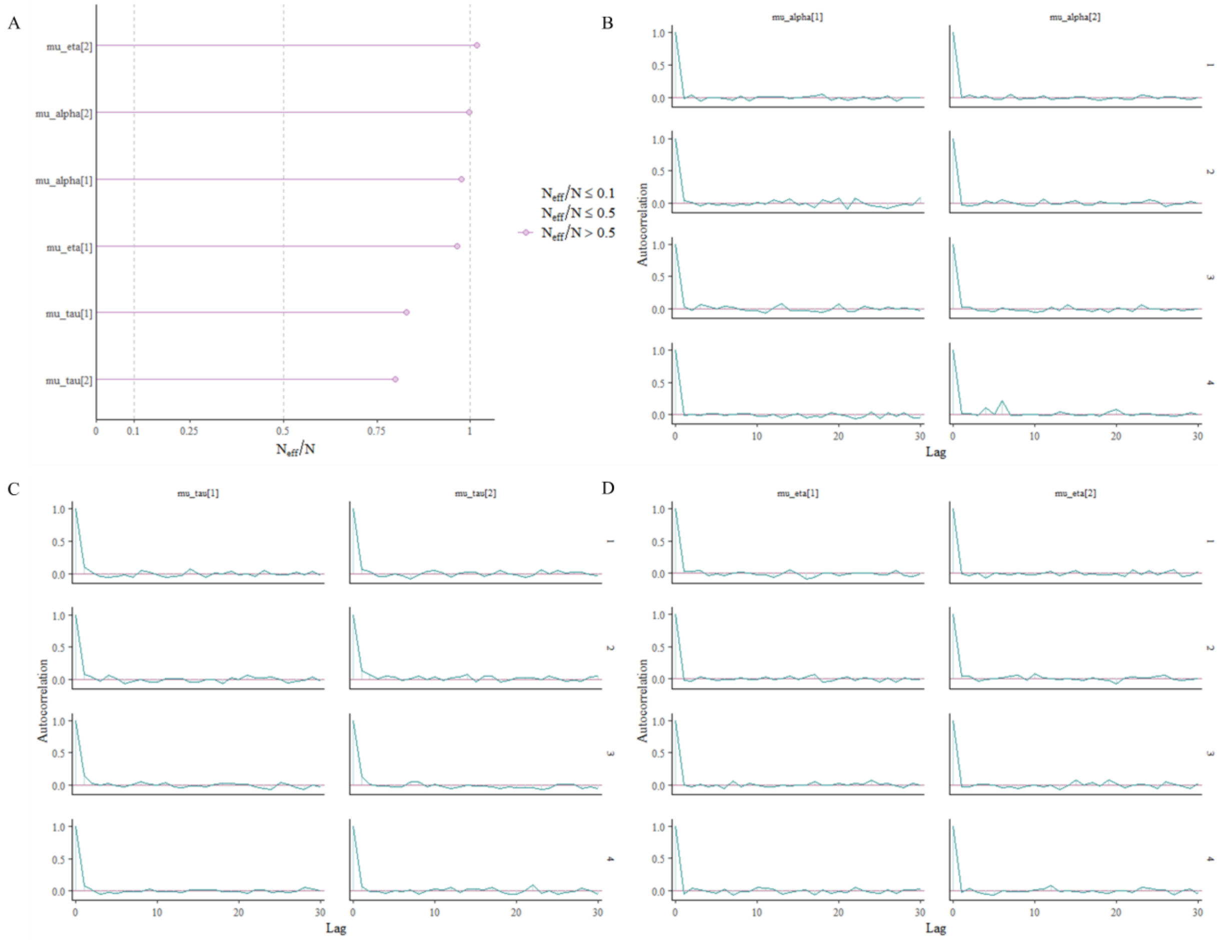

Note. Figure S16A (‘mcmc_neff’) depicts in the plot points that represent the neff/N ratio, the larger the ratio neff to N is the better (65). Same conventions as in Figure S11.

Figure S 18. Individual-level posterior distributions (medians + 95% HDI) among paraments in Study 1 ('student sample') females only

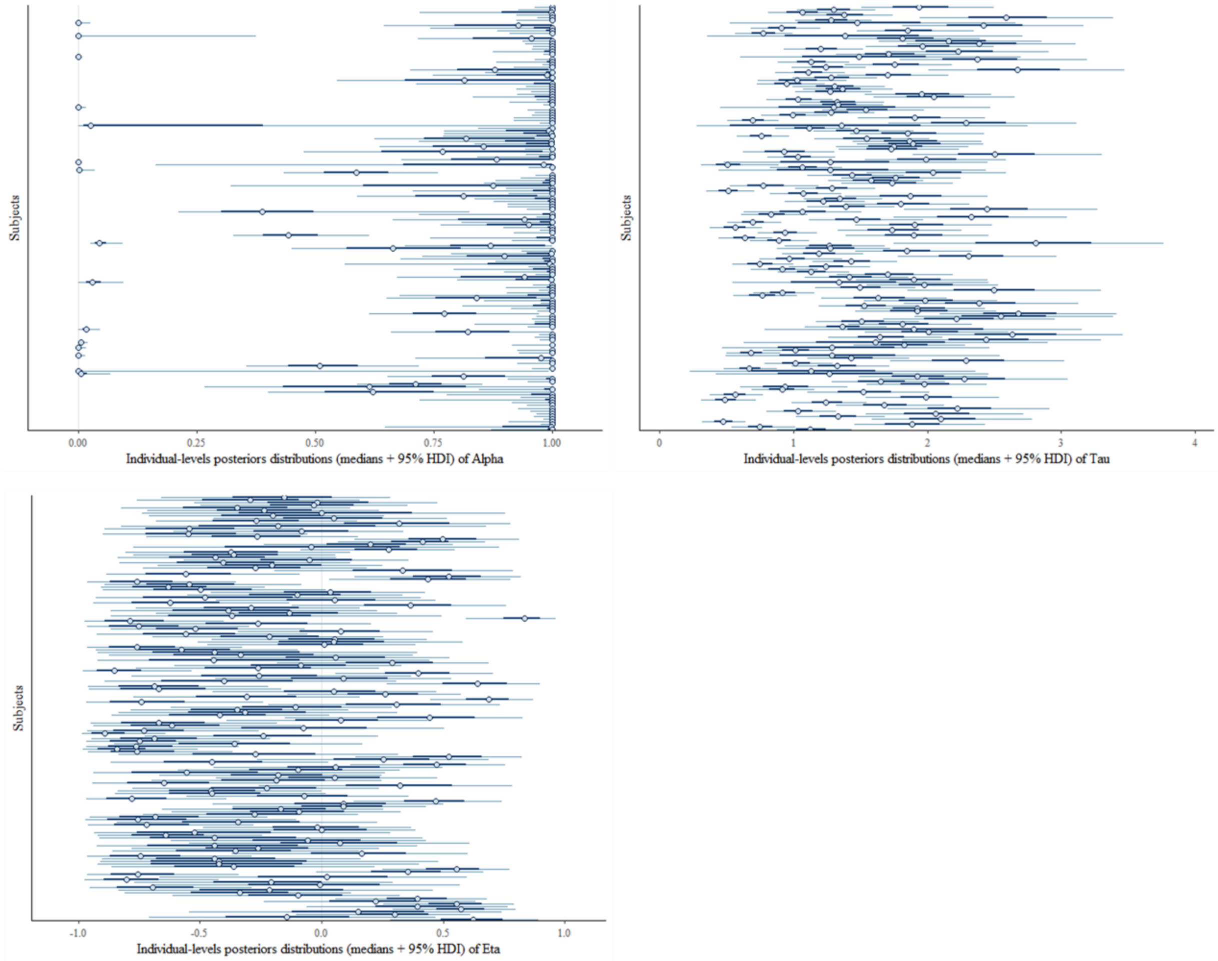

Note. Same conventions as in Figure S13.

Figure S 19. Population-level parameters from Study 1 – females only sample.

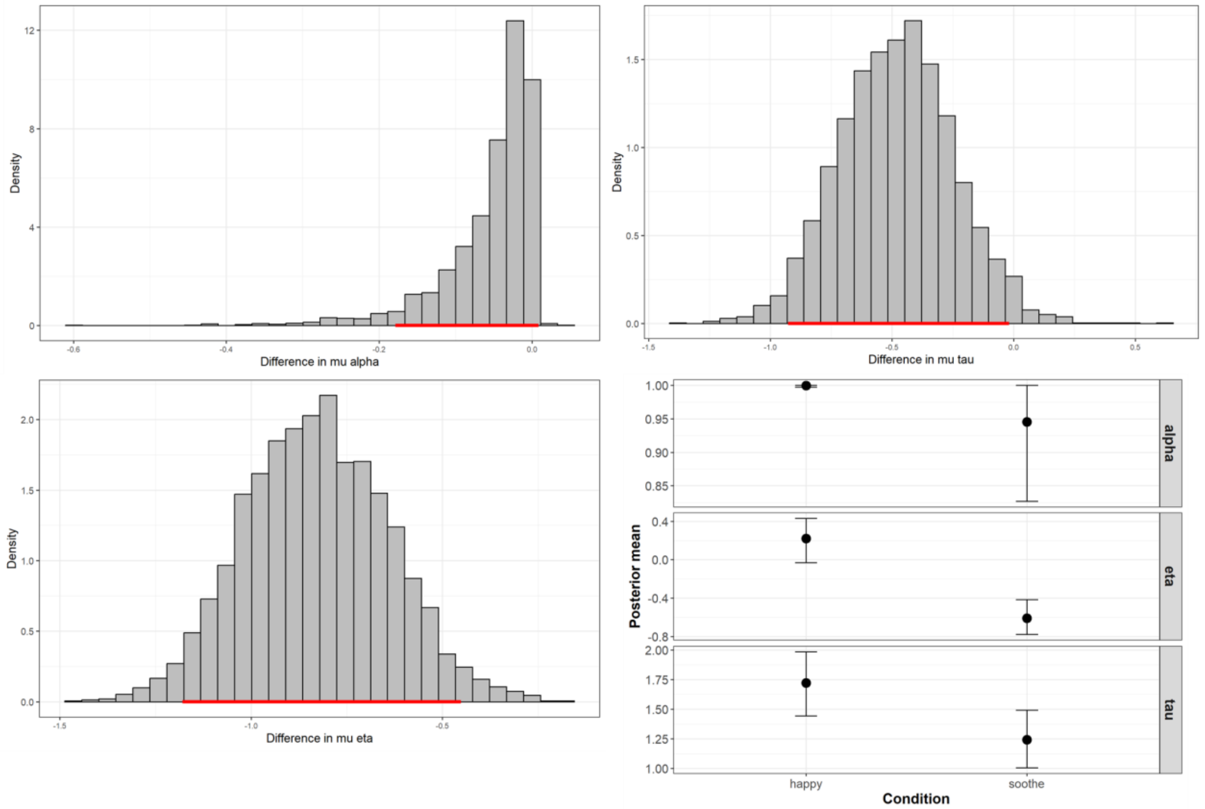

Note. Difference in population-level posterior distributions between the “Soothe the baby” and the “Keep the baby happy” conditions in the student (“Study 1”) sample - females only. The median (± 95% credible intervals) for the population-level means are shown in the bottom right panel.

2.8.2 Associations between mental health measures and parameters of interest

In evaluating the associations between mental health measures and our parameters of interest (N = 161), the results were mostly consistent, with 95% CrIs largely overlapping with the findings in the total student sample. Notably, the associations between higher personality difficulties, depressive symptoms, impulsivity and lower learning rates were slightly stronger (Table S 1). We also observed additional evidence for an association between higher impulsivity and lower learning rates. When evaluating decision-making strategies (balance between exploration and exploitation), we found little evidence that

higher state anxiety was associated with higher exploration, thus resulting in lower exploitation (-0.07, 95%CrI: -0.12 to 0.02).

Table S 1. This table reports the median posterior effect size and 95% CrI representing the association between levels of personality difficulties, depression, anxiety and impulsivity and the parameters of interest in Study 1 (only in females).

Study 1 (Nulliparous participants – females only)					
**Mental health measure[1]**	Parameters[2]	Number of participants	Estimate (Median posterior distribution)	95% CrI	Rhat
	**Alpha**				
SAPAS (personality difficulties)		161	-0.12	-0.28 to 0.05	1.00
EPDS (depression)		-	-	-	-
EPDS (depression without anxiety items)		-	-	-	-
MFQ (depression)		161	-0.11	-0.27 to 0.06	1.00
STAI-Y1 (State anxiety)		161	-0.06	-0.21 to 0.09	1.00
STAI-Y2 (Trait anxiety)		161	-0.04	-0.20 to 0.12	1.00
BIS-11 (Impulsivity)		161	-0.11	-0.25 to 0.03	1.00
	**Tau**				
SAPAS (personality difficulties)		161	-0.03	-0.08 to 0.02	1.00
EPDS (depression)		-	-	-	-
EPDS (depression without anxiety items)		-	-	-	-
MFQ (depression)		161	0.01	-0.07 to 0.04	1.00
STAI-Y1 (State anxiety)		161	-0.07	-0.12 to -0.02	1.00
STAI-Y2 (Trait anxiety)		161	-0.01	-0.06 to 0.05	1.00
BIS-11 (Impulsivity)		161	-0.02	-0.08 to 0.03	1.00
	**Eta**				
SAPAS (personality difficulties)		161	0.08	-0.01 to 0.17	1.00
EPDS (depression)		-	-	-	-
EPDS (depression without anxiety items)		-	-	-	-
MFQ (depression)		161	-0.02	-0.12 to 0.08	1.00
STAI-Y1 (State anxiety)		161	0.03	-0.06 to 0.12	1.00
STAI-Y2 (Trait anxiety)		161	0.03	-0.06 to 0.13	1.00
BIS-11 (Impulsivity)		161	-0.10	-0.20 to -0.01	1.00

References

Aylward, J., Valton, V., Ahn, W.-Y., Bond, R. L., Dayan, P., Roiser, J. P., & Robinson, O. J. (2019). Altered learning under uncertainty in unmedicated mood and anxiety disorders. *Nature Human Behaviour*, *3*(10), 1116–1123. https://doi.org/10.1038/s41562-019-0628-0

Barratt, E. S. (2007). *Barratt Impulsiveness Scale 11 (BIS-11) 1995*.

Gelman, A. (2006). Prior distributions for variance parameters in hierarchical models (comment on article by Browne and Draper). *Bayesian Analysis*, *1*(3), 515–534.

Gelman, A., Carlin, J. B., Stern, H. S., Dunson, D. B., Vehtari, A., & Rubin, D. B. (2013). *Bayesian Data Analysis* (Chapman & Hall/CRC Press, Ed.; third edit).

Germans, S., Van Heck, G. L., & Hodiamont, P. P. G. (2012). Results of the search for personality disorder screening tools: clinical implications. *The Journal of Clinical Psychiatry*, *73*(2), 18226.

Messer, S. C., Angold, A., Costello, E. J., & Loeber, R. (1995). Development of a short questionnaire for use in epidemiological studies of depression in children and adolescents: Factor composition and structure across development. *International Journal of Methods in Psychiatric Research*.

Moran, P., Leese, M., Lee, T., Walters, P., Thornicroft, G., & Mann, A. (2003). Standardised Assessment of Personality–Abbreviated Scale (SAPAS): preliminary validation of a brief screen for personality disorder. *The British Journal of Psychiatry*, *183*(3), 228–232.

Mukherjee, D., Filipowicz, A. L. S., Vo, K., Satterthwaite, T. D., & Kable, J. W. (2020). Reward and punishment reversal-learning in major depressive disorder. *Journal of Abnormal Psychology*, *129*(8), 810–823. https://doi.org/10.1037/abn0000641

Murray, D., & Cox, J. L. (1990). Screening for depression during pregnancy with the edinburgh depression scale (EDDS). *Journal of Reproductive and Infant Psychology*, *8*(2), 99–107. https://doi.org/10.1080/02646839008403615

Rescorla and Wagner. (1972). A theory of Pavlovian conditioning: Variations in the effectiveness of reinforcement and nonreinforcement. *Current Research and Theory*, 64–99.

Spielberger, C. D. (1983). *State-trait anxiety inventory for adults*.

Stan Development Team. (2022). *Stan User's Guide Version 2.31*. https://mc-stan.org/docs/reference-manual/effective-sample-size.html#ref-Geyer:2011

Sutton, R. S., & Barto, A. G. (2018). *Reinforcement learning: An introduction*. MIT press.

The MathWorks Inc. (2022). *Optimization Toolbox version: 9.4 (R2022b)* (9.4). The MathWorks Inc.

Vandekerckhove, J., Tuerlinckx, F., & Lee, M. D. (2011). Hierarchical diffusion models for two-choice response times. *Psychological Methods*, *16*(1), 44.
